# The Layer-Oriented Approach to Declarative Languages for Biological Modeling

**DOI:** 10.1371/journal.pcbi.1002521

**Published:** 2012-05-17

**Authors:** Ivan Raikov, Erik De Schutter

**Affiliations:** 1 Okinawa Institute of Science and Technology, Onna-son, Okinawa, Japan; 2 University of Antwerp, Antwerp, Belgium; Ecole Polytechnique Fédérale de Lausanne, Switzerland

## Abstract

We present a new approach to modeling languages for computational biology, which we call the layer-oriented approach. The approach stems from the observation that many diverse biological phenomena are described using a small set of mathematical formalisms (e.g. differential equations), while at the same time different domains and subdomains of computational biology require that models are structured according to the accepted terminology and classification of that domain. Our approach uses distinct semantic layers to represent the domain-specific biological concepts and the underlying mathematical formalisms. Additional functionality can be transparently added to the language by adding more layers. This approach is specifically concerned with declarative languages, and throughout the paper we note some of the limitations inherent to declarative approaches. The layer-oriented approach is a way to specify explicitly how high-level biological modeling concepts are mapped to a computational representation, while abstracting away details of particular programming languages and simulation environments. To illustrate this process, we define an example language for describing models of ionic currents, and use a general mathematical notation for semantic transformations to show how to generate model simulation code for various simulation environments. We use the example language to describe a Purkinje neuron model and demonstrate how the layer-oriented approach can be used for solving several practical issues of computational neuroscience model development. We discuss the advantages and limitations of the approach in comparison with other modeling language efforts in the domain of computational biology and outline some principles for extensible, flexible modeling language design. We conclude by describing in detail the semantic transformations defined for our language.

## Author Summary

The pursuit for understanding of neural function by computational modeling has produced a variety of software tools, with each tool targeting specific audiences and often requiring input in its own distinct language. Consequently, comprehending and communicating neuroscience models is a difficult and time-consuming task. In this paper we suggest a new approach towards designing biological modeling languages, which we call the layer-oriented approach. The approach stems from the observation that diverse biological phenomena are described using a small set of mathematical formalisms (e.g. differential equations), which are structured according to some biological principles. Our proposal is illustrated by means of a computer language for describing computational models of ionic currents. The language consists of rules for expressing mathematical equations as well as rules to organize these equations according to the specific terminology used by neuroscientists. The layer-oriented approach offers two chief advantages. First, it allows the flexible use of mathematical equations to represent many different kinds of biological models. Second, it restricts the language within a framework of biological concepts so that existing modeling software can be reused. The goal of the layer-oriented approach is to help define appropriate notations for computational biology while enabling interoperability of software for biological modeling.

## Introduction

Scientists who construct computational models of biological processes often find it necessary to use several different software tools in order to carry out various forms of data analysis and model simulation. However, each tool may employ its own model description format, consisting of diverse syntactic structures, and often can make implicit assumptions that are not reflected in the corresponding technical documentation [1], [2]. As a result, constructing an exact implementation of a published model is a complex and time-consuming task.

As an example, in computational neuroscience, both the GENESIS [3] and NEURON [4] simulators provide a parameterized form of the Hodgkin-Huxley model [5] as a basic object for model construction, but with some important differences between their description languages. The Hodgkin-Huxley object that exists in the Genesis language allows the rate equations to be specified in functional form and thus it can express not only the standard formulation of the model, but a whole family of conductance-based models of ionic currents. The NEURON HOC language also provides a Hodgkin-Huxley object, but its rate equations are fixed and it only allows different values for the parameters and initial states. NEURON includes a separate language, NMODL, which is intended for detailed descriptions of ionic current mechanisms that are distinct from the Hodgkin-Huxley equations. Hence, the two simulators have very different assumptions about what is meant by a “Hodgkin-Huxley model”.

In

These efforts are now facing their own information exchange challenges [12]. For instance, the Simulation Experiment Description Markup Language (SED-ML) [13], which is an emerging standard for encoding numerical simulation protocols on top of SBML and CellML, has faced problems such as different sets of mathematical expressions allowed in different modeling languages and representing a diverse range of simulation time courses in the simulator software [14]. Other limitations of existing markup languages for biological modeling are pointed out in Section Discussion.

These issues suggest that a more comprehensive approach may be necessary to build an interoperable ‘stack’ of extensible declarative languages for model description, simulation protocols, data analysis and so on.

The layer-oriented approach described in this paper is a methodology to specify the syntax and semantics of several interlinked declarative languages (or layers), each targeted at a particular problem domain, and formally describe how they relate to one another. We refer to syntax as the grammar according to which the sentences of a language are constructed; semantics is the system of rules that gives meaning to those sentences. The layers are not standalone languages, such as in the case of SED-ML, SBML and CellML, but share common properties in order to ensure their compatibility.

The work presented here was developed prior to the authors' involvement in the NineML effort, which is a model description language developed as part of the Large-Scale Network Modeling initiative of the International Neuroinformatics Coordinating Facility (http://www.incf.org/) [15]. The design of NineML is also divided in semantic layers, however its focus is on describing large-scale networks of integrate-and-fire neurons, and its design significantly diverges from the language presented here, which is oriented towards conductance-based models of ionic currents.

The rest of this paper is structured as follows. Section Results gives an informal introduction to an example language for describing ionic currents, presents a high-level overview of the layer-oriented design of the language and highlights several language features necessary to express a complex model of currents in the Purkinje neuron. Section Discussion relates the layer-oriented approach to other model description language efforts and discusses its advantages and limitations. Section Methods presents a detailed syntactic and semantic specification of all layers in the example language and includes a brief summary of pertinent computer science literature.

## Results

We propose the layer-oriented approach as a methodology to develop common semantics for declarative biological modeling languages and supporting software tools. The premise of the approach is that computational models of biology are not merely a flat collection of equations, but follow a hierarchical structure that reflects the organization of the actual biological object or process [16].

This work was initially motivated by our attempts to express models of Purkinje neuron currents in a declarative format and to solve the problem of automatically merging together ionic current mechanism descriptions in the NMODL language to reduce simulation run time (see Section Ionic current mechanism mapping problem in NEURON).

Implementing the necessary model description concepts in an equation-based framework while preserving the neuroscience-specific model structure led us to adopt a general layer-oriented approach, where neuroscience-specific concepts are explicitly mapped onto structured equations. As a result, this approach accommodates a number of additional modeling concepts, supports multiple code generation targets and further offers several advantages:

1. Semantic unambiguity: every element of a layer-oriented language has exactly one unique mathematical representation.
2. Extensibility: new elements and corresponding semantics can be added to layer-oriented language in a consistent and unambiguous manner.
3. Expressiveness: all possible relationships among the entities in a model of a biological system can be described.

A key assumption of the layer-oriented approach is that the target domain of modeling is sufficiently well-defined so that its concepts can be encoded using the methodology we outline. Thus, the approach might not be necessarily suited for modeling techniques that rely on empirical algorithms, as opposed to a well-understood mathematical theory. This limitation and a possible methodology for community development of a layer-oriented language are discussed in Section When and how to use the layer-oriented approach.

Furthermore, we emphasize that our approach is concerned specifically with declarative languages. Variations of the layer-oriented approach based on algorithmic languages do exist in computational neuroscience. In Section Declarative and Algorithmic Languages we discuss this distinction and its implications. The introduction of Section Methods relates our approach to computer science literature on domain-specific language design.

### The layer-oriented approach by example

We first informally illustrate the layer-oriented approach with an example language for describing conductance-based ionic current models. Some technical details are omitted here, but complete formal grammar and semantic rules for the language are given in Section Methods. In the following sections we show how to use this language to describe a complex model of ionic currents in the Purkinje neuron.

The example language provides convenient idioms for common neuroscience modeling concepts. The layer-oriented approach ensures that each language idiom has a consistent mathematical representation that can be understood by each simulation or analysis software we desire to use. Furthermore, we will be able to extend the language by defining new concepts in terms of differential equations and other mathematical abstractions.

We begin with a representation of a Hodgkin-Huxley-style model, which implicitly relies on several physiological modeling concepts such as Ohmic currents and gating variables.

For the reader interested in technical details, the syntax presented below uses SXML, an alternative XML Infoset implementation based on Lisp s-expressions [17]. This syntax has an exact equivalent in conventional XML, but the use of s-expressions eliminates the necessity of closing tags and considerably reduces syntactic clutter.

(Membrane-potential

 (Membrane-capacitance 1.0 uF/cm*cm)

 (Ohmic-current Na (E = 115 mV) (g_max = 120 mS/cm*cm)

  (gating m (power 3)

   (forward-rate (2.5 - 0.1*V)/((exp (2.5 - 0.1*V)) - 1))

   (reverse-rate (0.125 * exp(-V/80))))

  (gating h (power 1)

   (forward-rate …)

   (reverse-rate …))

) ;; end of Ohmic-current Na

(Ohmic-current K (E = …) (g_max = …)

  (gating n (power 4)

   (forward-rate …)

   (reverse-rate …))

) ;; end of Ohmic-current K

 (Ohmic-current Leak …)

) ;; end of Membrane-potential

Although the sentences above are a fairly idiomatic representation of the Hodgkin-Huxley model, we must ensure that the underlying mathematics are consistently represented when this model is loaded in different software environments.

To meet this requirement we need a language mechanism to automatically transform the above model code into the corresponding equations:

Capacitance = 1.0 uF/cm*cm

g_Na = g_max_Na * m_Na * m_Na * m_Na * h_Na

I_Na = g_NA * (V - E_Na)

dm_Na/dt = alpha_m_Na(V) * (1 - m_Na) - beta_m_Na * m_Na

alpha_m (V) = (2.5 - 0.1*V)/((exp (2.5 - 0.1*V)) - 1)

…

V = - (I_Na+I_K+…)/Capacitance

**Figure 1:**
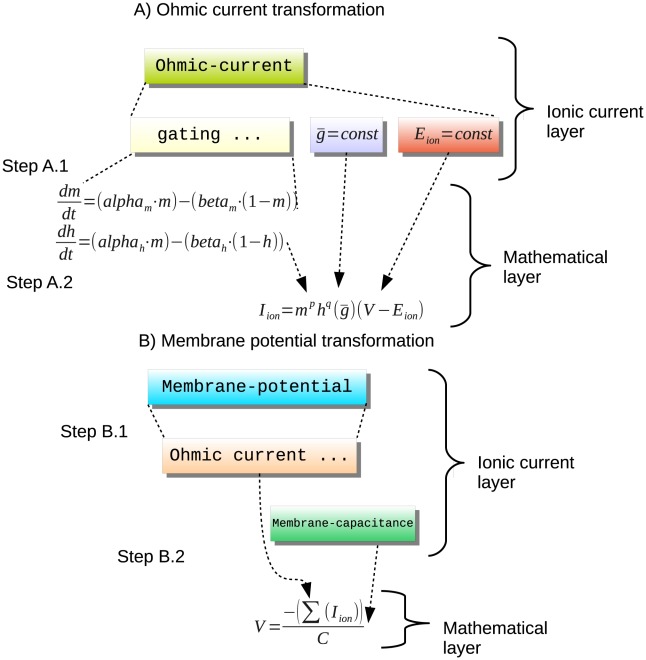
Conceptual overview of the transformation steps involved in generating a membrane potential equation from a collection of Ohmic current definitions. Step A.1 obtains the constituent parts of the gating component and constructs the gating dynamics equations. Step A.2 constructs the ionic current equation using the gating variables, maximal conductance and reversal potential declarations. Step B.1 assembles together all Ohmic current declarations and Step B.2 is constructing the membrane potential equation.

Figure 1 is a conceptual overview of the steps performed by such a transformation mechanism in order to construct ionic current and membrane potential equations. In step A.1 the gating variable declarations are used to construct the gating dynamics equation, and in step A.2 the maximal conductance and reversal potential declarations are combined together to form the complete ionic current equation. In step B.1 all Ohmic current declarations are assembled together and used to construct the membrane potential equation in step B.2.

**Figure 2:**
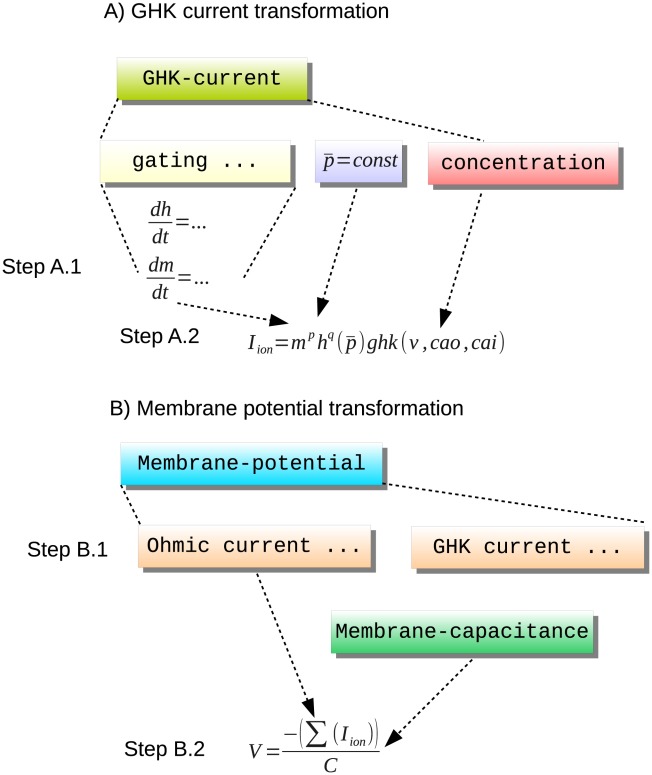
Conceptual overview of the transformation steps involved in generating a membrane potential equation from a collection of Ohmic and GHK current definitions. Step A.1 constructs the gating dynamics equations and extracting the maximal permeability and concentration definition. Step A.2 constructs the ionic current equation using the gating values, the maximal permeability and the GHK equation, which depends on the definitions of concentration. Step B.1 assembles together all Ohmic and GHK current declarations and Step B.2 constructs the membrane potential equation.

This kind of transformation mechanism is key to ensuring consistency of the mathematical representations of our model. Furthermore, extending the set of available model description concepts then becomes a matter of defining appropriate transformation rules. For example, to accommodate Goldman-Hodgkin-Katz (GHK) currents we use the transformation rules illustrated in Figure 2. This example already demonstrates the extensibility of the layer-oriented approach. Figure 2 clearly shows that incorporating this important feature requires only minimal extensions to the structures presented in Figure 1. Note that the gating mechanisms are identical for Figures 1A and 2A and that Figure 2B just adds GHK currents at the appropriate structure without disturbing the overall model structure.Analogously with the Ohmic current transformation, step A.1 constructs the gating dynamics and step A.2 constructs the GHK current equation.

Thus, the layer-oriented approach is primarily concerned with definitions of biological modeling concepts and their equivalent equational form. The transformation from one to the other is explained in detail in the following sections.

### Concepts of the layer-oriented approach

The layer-oriented approach is a structured methodology to define notations for declarative computational models. It involves:

- *language layers*, which are collections of grammatical rules that correspond to concepts from a particular domain, such as computational neuroscience or differential calculus;
- *semantic transformation functions*, which assign semantics to the layers, in the form of rules that specify how concepts from one layer can be represented by a combination of concepts in another layer.

The question of which biological modeling and mathematical concepts are chosen and grouped in layers is one that must be properly answered by the scientific community. The layer-oriented approach provides the technical means to formalize the relationships between the domains of biological modeling and mathematical concepts. The process of formalizing these relationships is a way to identify and eliminate potential flaws in the language and to communicate the language semantics in a concise manner. More on this topic can be found in Section When and how to use the layer-oriented approach.

**Figure 3:**
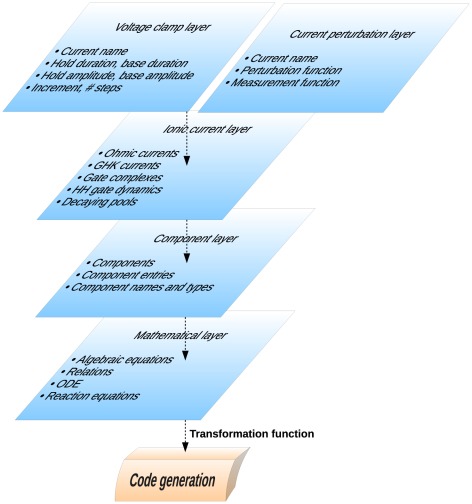
Conceptual layers of modeling and their relationships.

As a concrete example, Figure 3 illustrates the structure and the relationships of several language layers that together can describe the structure of computational neuroscience models of ionic currents as well as voltage clamp protocols, explained below.

The ionic current layer consists of elements that correspond to neuroscience modeling concepts, such as channel gates and ionic conductances. The mathematical layer consists of elements that correspond to general mathematical concepts, such as rate equations and functions.

Figure 3 is not meant to be an exhaustive representation of the biological modeling ‘universe’. It can be easily conceived that e.g. adding stochastic differential equations to the mathematical layer will allow a range of stochastic models to be included in the higher layers. The point is that the layer-oriented approach enables such additions to be explicitly and clearly specified while preserving full compatibility with existing definitions, as illustrated with the GHK example in Figure 2.

### A metalanguage for describing layers and transformations between them

In our approach, a semantic transformation function is a collection of rules that specify how concepts from one layer are represented as a combination of concepts from another. An important practical aim is to represent the layers and the transformations between them by means of a mathematical notation that does not have the clutter of programming details inherent to a concrete implementation yet can be easily expressed in any reasonable programming language.

Thus, the semantic transformation functions in this paper are written in a metalanguage that contains the essence of some typical patterns of programming languages. With this approach, the semantics of layer-oriented language can be described independently of the implementation language by a sequence of various layer transformation functions, e.g.:
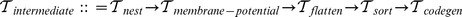
The sequence of transformation functions comprising 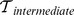 describes a set of common operations necessary to express a model of ionic currents as an environment of equations conforming to the syntax of the equation-oriented mathematical layer (detailed definitions are given in Section Methods).

The specification of a layer-oriented language then takes the form of semantic transformation functions for all layers, which can be straightforwardly mapped to an implementation. We note here that the metalanguage is not concerned with issues such as error handling for invalid input as these are details unique to each implementation.

A further practical benefit of this manner of specification is that semantic transformation functions provide a convenient blueprint for code generation, or the process of transforming computational biology models to computer-executable form [18], in this specific instance generation of Matlab or NMODL language. The two sequences of transformation functions below describe code generation for two very different software platforms (Matlab and the NEURON simulator) using largely identical sequences of steps (details are given in Section Methods).
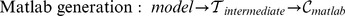


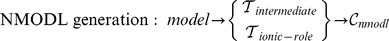



From a practical standpoint, 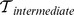 and its constituent parts need only be implemented once and reside in a standard software library, which can then be shared between multiple simulators and other software that aims to read this particular model description language. Additional information for code generation, such as provided by 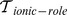 (needed for NEURON) can also be specified with semantic transformation functions and implemented either as part of the standard library or for specific platforms.

The transformations specific to neuroscience modeling software are briefly described in the following sections. All transformation functions mentioned in this section are defined in Section Methods

### Components and structured layer-based models of ionic currents

Our model examples thus far have included the use of two layers, one for ionic current descriptions and one for equations and functions. The equation layer omitted any of the structure associated with biological interpretation of the equations, such as the gating elements. But the language must have the capability not merely to represent a set of equations, but to group related definitions and equations across layers.

We therefore introduce the notion of model components, which encapsulate related equations and functions that are part of a model. They are generic entities that are not concerned with how these equations are grouped together and permit arbitrary nesting of sub-components. We further characterize a component by its type and output quantities.

From the point of view of biological modeling, only particular combinations of nesting are valid. Wimalaratne et al. observed that allowing arbitrary structuring of hierarchical biological models leads to difficulties in model exchange, and therefore we need to define rules that require models of ionic currents to be structured according to the accepted principles of computational biology [19].

The syntax and semantics of the component layer and structuring rules are given in Section Methods. These rules stipulate that the following structure must be followed:

(Membrane-potential Modelname

 (Membrane-capacitance (out C)

  … definition of capacitance …)

 (Ohmic-current (name ion)

  (gating (out m)

   … equations for channel gate dynamics …)

  (pore (out gbar)

   … equations and parameters of maximal conductance …)

  (permeating-ion (name ion) (out e)

   … definition of reversal potential …)))

Compared with the previous example, the model structure above explicitly labels the sub-components of the ohmic current component (gating, pore and permeating-ion). While slightly more verbose, this notation allows easier formulation of transformation rules, as we explain in Section Methods.

These are not intended to be authoritative rules, but an illustration of the capabilities of the layer-oriented approach. A different set of rules can be easily formulated and formalized as determined by discourse in the scientific community. Further details can be found in Section Discussion and Section Methods.

### The Khaliq-Raman model of the cerebellar Purkinje neuron

**Figure 4:**
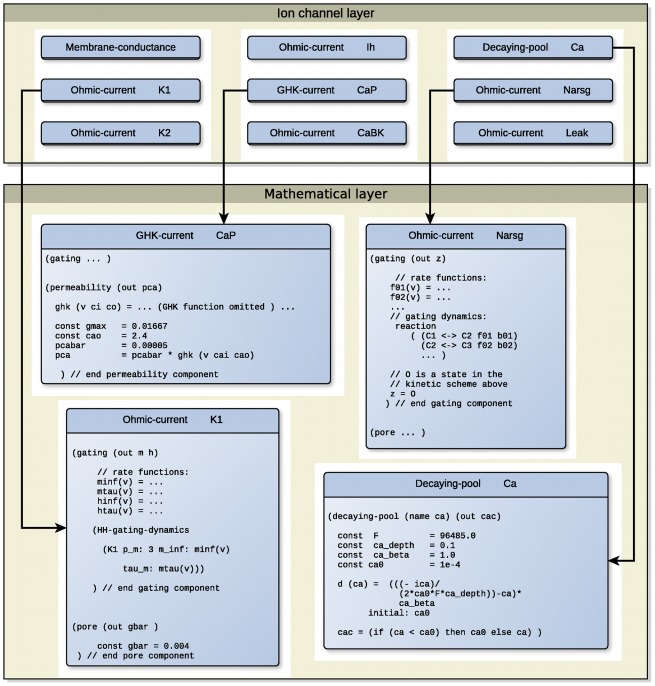
Components of a layer-oriented implementation of the Khaliq-Raman model of the Purkinje neuron. The model representation in the ionic current layer consists of a set of components that refer to the various biological concepts in the model — membrane capacitance, calcium concentration dynamics and the ionic currents comprising this model. Each component contains mathematical definitions pertaining to those biological concepts.

We have used the prototype language to implement a previously published model of the Purkinje neuron. The component abstraction gives us the ability to construct models as aggregations of components containing definitions of ionic gates, conductances and so on. Figure 4 illustrates the component structure of our description of the Khaliq-Raman model of cerebellar Purkinje neurons (ModelDB accession number 48332) [20]. The complete prototype listing is given in Supporting Text S1.

Our layer-oriented description of this model consists of the Ohmic and GHK current components already mentioned as well as a calcium concentration dynamics components that will be explained in the following subsections.

#### Parametric gating dynamics

The declarations contained in the Ohmic-current component shown in Figure 4 define four relations that represent activation and inactivation rates and whose expression bodies are omitted from the figure for brevity. The construct HH-gating-dynamics is a template which is expanded into equations for the two state variables m and h using the given rate function expressions.

Templates are special case of components where the contained equations are required to have certain names. A regular component can contain equations and functions with arbitrary names, but the HH-gating-dynamics template must contain equations that are exactly called m_inf, tau_m, etc. In all other aspects, templates are treated in the same manner as the other types of components.

The transformation function for HH-gating-dynamics is given in Section Methods.

#### Resurgent sodium current

Although the definitions shown above refer to standard Hodgkin-Huxley-type models, arbitrary reaction rules to represent gate dynamics can be included in the model as long as the correct component interface is used. Moreover, models of additional biophysical processes can be seamlessly incorporated in the functions that compute the channel opening and closing rates.

The resurgent sodium current in the Khaliq-Raman model uses a 13-state kinetic scheme and therefore we must use the Reaction type of equation, rather than the HH-gating-dynamics template. The transformation procedures for the lower-level layers already handle kinetic schemes, as shown in Section Methods and therefore we can represent this type of current without further extensions to the language. A fragment of the resurgent sodium kinetic scheme is shown in the Narsg component box in Figure 4.

#### P-type calcium current

The Khaliq-Raman model uses the standard Ohmic equation to describe most of its currents and the Goldman-Hodgkin-Katz (GHK) constant field equation [21] to describe its P-type calcium current. As illustrated in the introductory example, this has necessitated the addition of a GHK current transformation function and extending the membrane potential transformation function with an additional clause, following the GHK formulation [22].

The definition of the GHK current transformation function is given in Section Methods. It still refers to probability that channel gates are open, however the current equation now refers to a permeability quantity 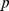 and no longer includes reversal potential. We have extended the set of component types with the type permeability and use it to encapsulate equations that compute current rather than conductance.

The CaP current can then be formulated by means of the permeability component, as shown in the the CaP component box in Figure 4.

In the code shown in the figure, the external calcium concentration is a constant but the internal concentration is given by variable cai, which does not appear to be defined in the component. As we will see in the next section, the internal concentration dynamics are defined in a separate component, which is not visible in the component defining the CaP current. In the present paper, we address this issue by a global declaration that specifies that the global identifier cai is related to the definitions in the calcium concentration component so that it is visible to declarations from other components:

input (cai from decaying-pool ca)

The above declaration specifies that the value cai must come from a component named ca of type decaying-pool. An example of such a component comes next.

#### Calcium concentration dynamics

Our description of the Khaliq-Raman model includes the component type decaying-pool, which is used to encapsulate the calcium concentration dynamics of the model. The representation of this component is the calcium decay equation due to Traub [23], shown in Figure 4.

The cac variable is exported from this component and, because of the input declaration in the previous section the semantic transformations described in Section Methods assign the value cac to the global cai.

### Simulation experiments

**Figure 5:**
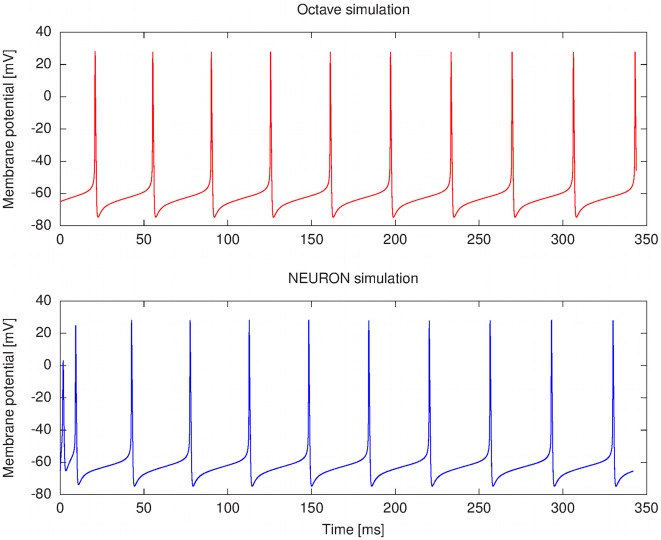
Comparison between simulation runs of the Khaliq-Raman model under different computing environments. NEURON is a software for simulations of neurons and networks of neurons. GNU Octave is an open-source equivalent to Matlab. The NEURON simulation was conducted with NEURON 7.1 using the cvode method, and the GNU Octave simulation was conducted with Octave 3.2 using the RADAU solver from the OdePkg toolbox version 0.6.10. In both cases, the software was run under Debian Linux 5.0 on a Dell Precision T5400 computer (CPU Intel Xeon E5430 2.66 GHz). The difference in how the respective simulation platforms compute the initial values for the resurgent sodium current causes the initial discrepancy between the two simulation runs.

**Figure 6:**
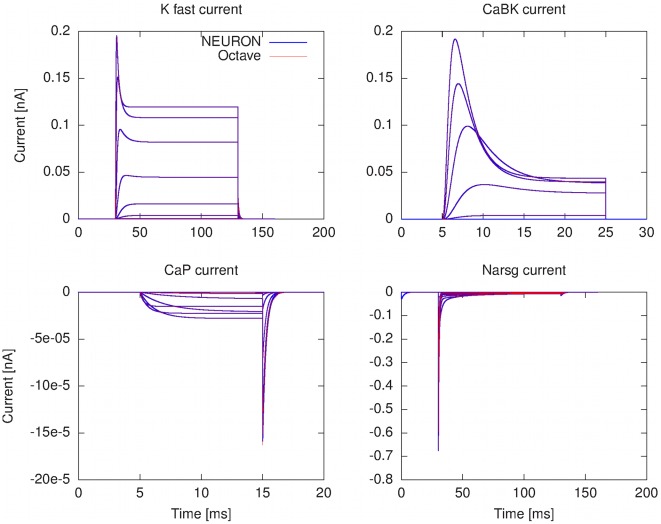
Comparison between voltage clamp simulation of the Khaliq-Raman model under different computing environments. The NEURON simulation was conducted with NEURON 7.1 using the cvode method, and the GNU Octave simulation was conducted with Octave 3.2 using the RADAU solver from the OdePkg toolbox version 0.6.10. In both cases, the software was run under Debian Linux 5.0 on a Dell Precision T5400 computer (CPU Intel Xeon E5430 2.66 GHz).

The layer-oriented approach can be easily applied to describing simulation experiments. The simulation results in Figures 5 and 6 were produced from the same model description with code generated by our prototype implementation of a translator for layer-oriented neuroscience models, as applied to the Khaliq-Raman model. The simulation software used was NEURON 7.1 and GNU Octave 3.2, in both cases running under Debian Linux 5.0 on a Dell Precision T5400 workstation. The code generation algorithm is based on the transformation rules defined in Section Methods. Additional simulation results addressing runtime efficiency in the NEURON simulation environment are described in Section Ionic current mechanism mapping problem in NEURON and presented in Supporting Figure S1.

The transformation functions for simulation experiments are given in Section Methods. We define two new types of components, simulation and voltage-clamp, and use them to specify simulation and voltage clamp parameters for the different currents of the model, e.g.:

(simulation (out duration stepsize)

 (const duration = 2000)

 (const stepsize = 1e-4)

)

(voltage-clamp (name CaBK)

 (out hold base stepsize nsteps holding-duration base-duration)

 (const hold = −90)

 (const base = −40)

 (const stepsize = 10)

 (const nsteps = 5)

 (const holding-duration = 5)

 (const base-duration = 20))

The semantics associated with each component of type voltage-clamp require that there must be a corresponding ionic current component of the same name. This then allows the generation of voltage clamp scripts that are consistent with the currents of the model.

#### Ionic current mechanism mapping problem in NEURON

To aid the construction of models and simulations the NEURON simulation environment provides a number of predefined constructs that correspond to familiar neuroscience idioms. User-defined mechanisms, such as voltage- and ligand-gated ion channels, diffusion, buffering, etc., can be added to the default set of mechanisms by writing model descriptions in NMODL, an equation-oriented declarative language.

These mechanisms are often structured such that there is a one-to-one mapping between NMODL files (.mod extension) and ionic current mechanism descriptions (7 A).

**Figure 7:**
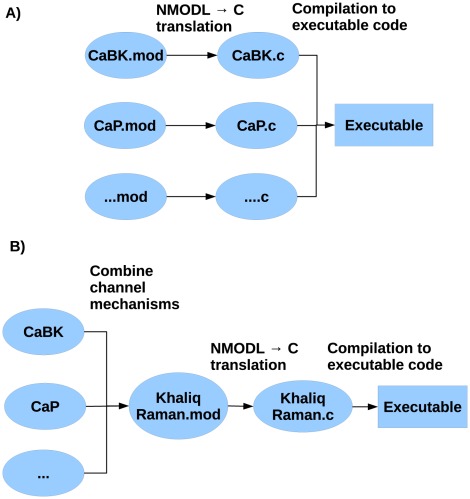
Ion channel mechanism mapping problem in NEURON. A) standard approach; B) merging of several ion channel mechanisms in order to improve efficiency. Our layer-oriented code generation tool generates the .mod files, the .c files are generated by NEURON.

However, it is frequently advantageous to combine the descriptions of several mechanisms in the same NMODL file so that their equations can be solved together, in order to improve the numerical efficiency of the simulation. Unfortunately, the standard NEURON software does not provide means to merge mechanism descriptions automatically and users are forced to maintain large NMODL files that are difficult to read and understand. Ideally, the modeling language must permit an easy-to-read model description that can be automatically transformed into efficient code (Figure 7 B).

The difficulty in combining the ionic current equations together comes from the fact that the same variable names may be used for equations that belong to different types of ionic currents. This is not a problem as long as these equations reside in separate NMODL files, however merging them together presents the risk of collisions between common variable names (e.g. 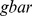) and therefore the users must resort to a very careful (and possibly verbose) coding style to ensure unique naming.

This issue is naturally solved by the modularity and semantic unambiguity of our approach because the equations for each ionic current reside in separate components and a renaming step in the transformation function ensures that each variable has a unique name that is prefixed by the name of the component (specified in detail in Section Methods). Furthermore, each ionic-current component contains information identifying the ionic species, which is used to combine the current equations for all channels of a given ionic species.

As a result, the NMODL code generator implemented in our prototype software allows a very systematic methodology for exploration of NEURON's performance. We have conducted a number of performance benchmarks with the Khaliq-Raman model, as detailed in Supporting Figure S1. The average simulation time using merged mechanism descriptions was reduced by 18.5% when using NEURON's variable time step solver and by 22.3% when using NEURON's fixed time step solver.

## Discussion

The shortcomings of existing standardization efforts suggest that future work should address the formal specification of mathematical concepts and the mapping of high-level modeling concepts to computational representation. This will make it possible to uniformly describe, share and use a new modeling technique within the same language. In particular, we believe that the layer-oriented approach has the following advantages over the existing approaches.

### Semantic unambiguity

The different domains and subdomains of computational biology each require that models are structured according to the accepted terminology and classification of that domain. Therefore, successful development of future biological modeling languages will depend on appropriately formalised representation of domain knowledge. One common approach to developing such formalizations are the multiple ontological efforts to represent various biological entities for multiple levels of granularity [24].

Our layer-oriented approach complements ontologies with the systematic development of domain-specific language rules so that the conventions and categories of the domain are distinctly and clearly represented to the user, while generality is preserved by the underlying layers that provide access to general mathematical and algorithmic concepts.

### Extensibility

By ‘extensibility’ we mean functionality to describe new modeling techniques in addition to those provided by standard model databases.

For example, suppose that a scientist wishes to use conditional expressions in the mathematical layer of a layer-based language, which is necessary for e.g. threshold detection in the integrate-and-fire formalism [25].

In such a case, the mathematical layer can be extended with conditional primitives to express transitions between dynamical systems and the neuroscience modeling layer can be extended with a regime concept, which encapsulates the subthreshold equations and specifies the firing condition and reset equation. The mapping between the high-level regime concept and the condition primitives can be defined by a semantic transformation function.

As another example, suppose that a scientist wishes to integrate morphological descriptions in a layer-based language. While the examples in this paper do not address geometric descriptions and partial differential equations the same transformation approaches can be applied to define complex surfaces and dynamics based on core abstractions for spatial PDEs.

Once the precise hierarchy of concepts and mathematical mechanisms are defined by the community, a layer-oriented language can allow scientists using the language to formally describe new approaches and make them shareable without having to alter the core language specification, as must be done for NeuroML.

### Expressiveness

As already observed by Wimalaratne, et al., explicitly defined hierarchical structuring rules are a necessity for many kinds of biological modeling. As we discuss in Section Existing biological modeling languages, some of the key evolutionary improvements in existing and emerging modeling languages are related to modularity, hierarchical structuring and expressing relationships between different components of a biological model. These properties are well-addressed in our approach by means of compositionality.

We refer as compositionality to the ability to compose a model from pre-existing parts. For example, given a set of standard ion channel objects from a model library and a set of parameters provided by the user, the equations for the model could be automatically constructed depending on the chosen channel objects.

A sophisticated component model is required to support descriptions such as a dendritic maximal conductance that is dependent on distance from the soma [26]. In order to support such functionality, the language must have formal semantics for composition and extension.

Layer-based components in a biological modeling language can express different functional and structural relationships and allow scientists to invent and share their own components, as well as build on the existing mechanisms.

### When and how to use the layer-oriented approach

One of the most important problems facing biological modeling languages is formulating the extent and requirements of the target domain. The layer-oriented approach provides the technical means to formalize the relationships between the domains of biological modeling and mathematical formalisms, but the researchers who wish to design and use such a language must already have some informal understanding of these relationships.

Once the domain is well-defined in terms of mathematical formalisms, as is the case with deterministic models of ionic currents in computational neuroscience, our layer-oriented approach can be applied by constructing a formal grammar for the language and corresponding transformation rules that explicitly link the biological modeling concepts to mathematical formalisms. As we show in Section Methods, the transformation rules can be written in a metalanguage that generalizes the typical patterns of programming languages without the operational details of a real implementation. The process of writing and understanding such rules assists researchers in clarifying and refining the semantics of the language, as Scott and Strachey showed in their influential work on programming language specification [27], [28].

Constructing a set of transformation rules for a given biological modeling concept may be a whole scientific endeavor, such as, for example, approximating the voltage dynamics of 3D cell membranes with the 1D cable formalism commonly used in computational neuroscience [29].

Furthermore, our approach relies on a mathematical language that is sufficiently rich to formulate all concepts and problems of the scientific field of interest. The development of computational science suggests that mathematical languages based on ODEs and PDEs are well-suited to express many theories and concepts of physics and chemistry. However, additional formalisms, such as stochastic equations, may be necessary to model problems in computational biology. The layer-oriented approach as a method for interoperability assumes that such additional formalisms would be consistently supported by several software platforms.

It is possible that for some biological concepts there exist semantic ambiguities, i.e. several alternative mathematical formulations. The layer-oriented approach is modular and can accommodate different sets of transformation rules for the same concepts in the form of namespaces or modules [30], but ultimately it is the responsibility of the language designers to use such technical tools to resolve the differences between the mathematical approaches.

The layer-oriented approach would not be applicable in a case where a biological modeling concept has only an empirical algorithmic representation and no consistent underlying mathematical theory. This is a consequence of the declarative nature of the approach. For example, the exact stochastic simulation algorithm (SSA) is widely used in computational biology [31]. However, the necessity to simulate every reaction event causes the algorithm to be too slow for some applications. An approximation strategy known as tau-leaping sacrifices exactness for reduced computational cost [32]. At present there is no widely adopted declarative generalization of SSA and tau-leaping, although proposals have been made [33]. Applying the layer-oriented approach to modeling problems based on tau-leaping, or other approximations of SSA, would require that the various decision procedures involved are represented in a declarative form that reflects the underlying mathematical model. In this sense our approach is limited by the scientific understanding of the concepts in the particular modeling domain.

Another important aspect of designing biological modeling language is the process of community validation. For instance, the community validation process of SBML Level 3 involves having at least two independent software implementations of a proposed feature before that feature can be considered for inclusion in the standard. From our personal observations on the development process of the emerging NineML language, the NineML committee has also converged on peer-reviewing implementation code as means to ensure that prototype implementations of the language not only have the same grammar, but also have consistent and community-approved semantics. However, the informal processes of SBML and NineML are limited by the fact that code in different programming languages cannot in general be directly compared. The layer-oriented approach is a way to lift this restriction. It enables the community first to agree on the semantic transformation rules, then to relate them to particular software implementation. It does not mandate a particular implementation, or a particular programming language, and thus can be used by a diverse community of developers. As a further step in language specification, the layer-oriented approach opens the possibility for using mathematical reasoning methodologies [34] to formally prove that a particular software implementation is faithful to a particular set of semantic transformation rules.

### Declarative and algorithmic languages

Variations of the layer-oriented approach are not new to computational neuroscience. The NEURON simulator has pioneered the use of an introspective interpreter (HOC or Python) and a declarative model description language (NMODL) for extending the available modeling mechanisms.

However, the work presented here is specifically concerned with layers of purely declarative languages. In our approach, the interfaces between layers are explicitly specified in an implementation-neutral mathematical notation and additional layers can be introduced in a consistent and conceptually clear manner. In contrast, simulators such as NEURON typically employ an algorithmic language for experiment control and a declarative language for model equations, and the details of interfacing the two languages are unique to the particular software implementation.

Declarative languages describe problems in a particular domain, and possibly some properties of the desired solutions, rather than explicit mechanisms for computing solutions [35], [36]. Algorithmic languages take the form of stepwise machine instructions for performing computation. Algorithmic languages have much greater expressive power than declarative ones, however they introduce operational details that might be entirely irrelevant to the higher-level concepts that they express.

Because of the expressiveness of algorithmic languages, it could be argued that all tasks in neuroscience simulation and modeling could be accomplished with a combination of NMODL and HOC or Python, or similar combination of declarative and algorithmic languages. However, the many engineering details of interfacing such languages – variable scoping, data representation and propagation, control flow – would make any such combination of languages unique and difficult to comprehend and to replicate in different software implementations.

Because in our approach each layer is declarative and constrained to a specific purpose, a complete set of rules can be given for how the different layers relate to one another and how executable code can be generated from a layer-based description. Such rules then provide a convenient blueprint for consistent and interoperable software implementations.

### Existing biological modeling languages

#### NeuroML

The primary goals of the NeuroML family of modeling languages [6], [7] are ability to express commonly used concepts in computational neuroscience and support of a large number of published models. To achieve these goals, the NeuroML development team has been focused on defining language concepts that closely correspond to the modeling idioms used by existing and well-established simulators, such as NEURON and GENESIS.

However, a number of sweeping changes to the language were found necessary by the NeuroML team when it was decided to support the PSICS software [37] as a simulation platform. This restructuring revealed some weaknesses of NeuroML 1.x, which are consistent with our experiences with describing the Khaliq-Raman model using ChannelML versions 1.6.x and 1.7.1. We can summarize these weaknesses in the following categories:

1. Lack of formally-defined semantics for the elements of the language: The ChannelML 1.x standard does not give the precise mathematical definition of its concepts, nor how each concept relates to the other structures in the language. It merely states that a ‘ChannelType’ entity could contain an ‘hh_gate’ or a ‘ks_gate’, but the proper mathematical and/or algorithmic background is not given anywhere in the specification.
  The problem with this approach is that it is very difficult to ensure consistent semantics when transforming ChannelML models to code for particular simulators and analysis software, such as NEURON and XPP [38].
2. A lack of means to specify conductance and current laws: The lack of a mathematical model for ChannelML means that no support is provided for non-Ohmic definitions of conductance and current, such as the Goldman-Hodgkin-Katz formalism.

#### SBML and CellML

SBML [8], [9] and CellML [10] are two well-known XML-based model exchange formats used in the life sciences. SBML in particular is well supported by a wide array of software packages. SBML is designed so as to capture several biophysical concepts at the core language level. For example, chemical reactions and species are an integral part of the language core. SBML was originally developed for exchange of monolithic models between simulation programs and its Level 1 and 2 specifications did not support reusable model components [39]. SBML Level 3 is developed as a modular language and its specification is organized in a central core and extension packages layered on top of this core [8].

CellML version 1.1 is more abstract and provides a component abstraction with flexibility sufficient to model different types of biological concepts. A CellML component can be an entirely conceptual entity created for modeling convenience, or it can have some real physical interpretation (for example, it could represent the cell membrane). CellML allows several kinds of relationships between components to be expressed, such as containment or connectivity.

However, the component model of CellML 1.1 does not permit user-defined component relationships in a semantically meaningful way, nor does it support parametric components. Furthermore, the CellML specification offers few guidelines for how to produce well-structured, layered models. A mathematical model of a biological process can be represented in CellML in many different ways and the structure of a model mainly depends on the style of the individual author. Wimalaratne et al. have published guidelines for structuring CellML models, which encourage hierarchical structuring and reusable components with generic mathematical expressions [19]. But as the CellML specification and tools do not currently support the codification of these informal guidelines, the biophysical concepts isolated in one CellML model cannot be conveyed in a machine-readable format to the software that interprets this model.

#### InsilicoML

InsilicoML (ISML) is a language that can explicitly describe hierarchical structures of physiological functions in a mathematical model [40]. In ISML, each part of a model is called a module and relationships between modules are defined as edges. ISML is fully compatible with CellML 1.0 and adds features to annotate models with ontological information and links to model databases.

ISML features extensive support for spatial PDEs and its modularity features are an evolutionary improvement over CellML. Two unique features of ISML are the morphology and time series data types, which allow direct integration of models with experimental data.

Efforts such as ISML highlight the importance of layered semantic specification for biological modeling languages. In systems biology, models that integrate heterogeneous experimental data, which are stored in numerous life-science databases, can have considerable errors in data integration if different sources do not describe their information consistently [41]. Furthermore, a layer-oriented approach can be used to formally describe the features unique to ISML and incorporate them in other modeling languages and existing software.

#### PyNN

PyNN is a software package for simulator-independent specification of neuronal network models [42]. PyNN allows the users to write network model code in the Python programming language, and then run it without modification on any of the four simulators supported by PyNN.

The PyNN API is mainly aimed at describing populations of neurons and the connections between them. Neuronal dynamics are described not on the equation level, but are referenced via a library of standard neuron types. PyNN can express concepts commonly found in neuronal network modeling, however the exact computational semantics remain implicit in the targeted simulator platforms.

Reproducibility of results in different simulators can be achieved with PyNN only for those models that are a part of the pre-defined neuron library. Extending the repertoire of models supported by PyNN is achieved by implementing the necessary extensions in Python using specific internal APIs.

Our approach differs than that of PyNN in two ways. First, the layer-oriented language presented here is not a collection of models, but a collection of general concepts, such as Ohmic currents and differential equations, that can be then utilized to build particular models. This means that, as long as a model is expressible with these concepts, executable code can be automatically generated for this model by applying common semantic transformation rules and performing a final code generation step for the specific executable target.

Second, the specification of a layer-based model description language is intended to be independent of the semantics of the implementation language and thus our semantic transformation rules are given in a notation that is easy to encode in general-purpose programming languages, but is not biased towards a specific one. If, for example, one wished to implement some or all of the functionality of PyNN in a language such as Java, then they must find a way to translate the idioms of Python and the data structures and algorithms of PyNN to a suitable and idiomatic Java representation. Our semantic transformation rules rely on simple pattern match (destructuring), function call, list/set construction and decomposition. These are all operations present in the popular programming languages of today and thus it is possible for PyNN to accommodate our semantic transformation functions.

### Summary

Designing modeling languages involves the translation of the concepts of the domain into semantic concepts appropriate for computer representation. Often the transformation from domain-specific concepts into computer code cannot be done in a single step but requires several intermediate steps. The layer-oriented approach is an attempt to discern these intermediate semantic steps.

The layer-oriented approach relies heavily on model structuring. Structuring is a common modeling technique of dividing an object into a number of parts and indicating relationships between these parts. In this way quite naturally a layered model arises. Our prototype language defines a form of structuring based on components that allows models of arbitrary complexity to be constructed. In this way, the language provides extensibility and flexibility in describing new models that involve detailed biophysical modeling.

## Methods

Our approach is inspired by the work of Scott and Strachey on the mathematical foundations of the semantics of programming languages [27]. Scott and Strachey attempted to formalize and make explicit the meta-theories intuitively employed by language designers, and developed solid mathematical methods for language engineering [28]. Their approach resulted in clear, concise and unambiguous specification of programming language semantics and compiler transformations.

Among the innovations of Scott and Strachey was a calculus for semantic description in the form of a minimal metalanguage based on the lambda calculus [43]. Our metalanguage is based on a small subset of the Standard ML programming language [44] and is summarized in the following section.

While the present paper cannot include an exhaustive discussion of the software engineering methods for constructing a layer-oriented language, Paulson's “ML for the Working Programmer” [34] has a practical introduction on modeling domain concepts as mathematical objects, while Gunter's textbook on semantics [45] is an in-depth treatise on the mathematical foundations of programming languages.

From the point of view of domain-specific languages, our approach is most closely related to the pipeline pattern identified by Spinellis [46]. The pipeline pattern involves a chain of domain-specific language processors that are each dedicated to a specific sub-language. As Spinellis points out, “often a system can best be described using a family of DSLs,” and, “the use of the pipeline pattern encourages the division of responsibility among small specialised DSLs and discourages bloated feature-rich language designs.” Our approach refines the pipeline pattern in that all possible transformation paths are explicit, thus allowing a more rigorous process of validating and extending the language, but possibly at the loss of some flexibility.

### Metalanguage definitions

The following syntactic constructs are used in the metalanguage:
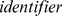


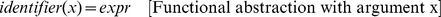


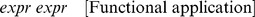


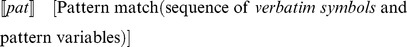
Expressions in the metalanguage are typically constructors for the various data structures that correspond to the domain-specific syntaxes discussed in this paper. For example, the definition
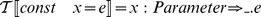
means that metafunction 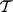 matches the sequence consisting of the symbol const, followed by the pattern variable 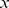 (which must be of a defined type), the symbol = and the pattern variable 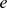. The result of the function is an entry constructed using the pattern variables and the Parameter constructor defined previously.

### An ionic current description language

The language we have developed for describing models of ionic currents has a hierarchical structure that is meant to reflect the logical relationships between the different parts of ionic current descriptions. For example, an Ohmic current consists of ionic current name, gating dynamics description and maximal conductance definition.

The syntax of this language in Backus-Naur form [47] is given below. We note that the definition of the Equation domain is not part of this language but refers to the equation-oriented language in the next section.
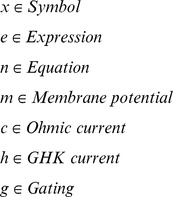


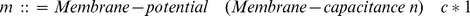


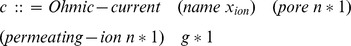


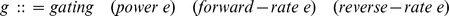



### An equation-oriented language

We base our layer-oriented approach on a domain-specific language that is capable of expressing differential and algebraic equations and later use it to construct complex models of ionic currents.

The language has a simple syntax for expressing relations and first-order differential equations, and we define a transformation function on this syntax that transforms every declaration in the language to an intermediate form suitable for further processing, such as code generation, or some type of model transformation, such as parameter perturbation.

The syntax of this language in Backus-Naur form is:
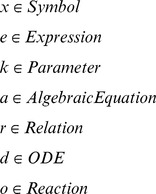


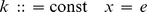
 [Constant during integration]


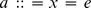
 [Algebraic equation]


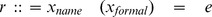
 [Relation]


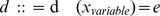
 [ODE of the form dx/dt = e]


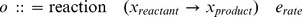
 [Forward kinetic scheme]


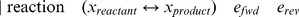
 [Forward and reverse kinetic scheme]

### Identifiers, entities, environments

The equation-oriented language is transformed to an intermediate semantic form suitable for further processing. We use an intermediate language of the following form:
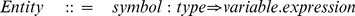
where

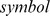
 is the identifier we use to refer to this entity;

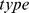
 is one of 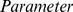, 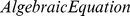, 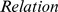, 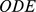, 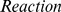

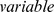
 represents the argument of a relation or the state variables in ODEs and reactions;

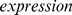
 is the right-hand side arithmetic expression

The transformation function 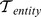 describes the process of creating new entities:
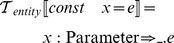


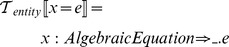


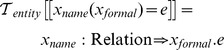


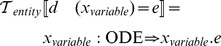


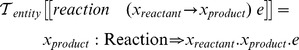



Entities are characterized by name, type and expression. However, we must use these entities together in order to solve the corresponding system of equations. We use an environment structure in which entities are indexed by name and type, and which can be queried to extract information for further model processing. We represent environments by a function
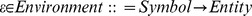
which we call the current environment of entities. We use the metafunction notation 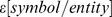 to express the extension of the current environment with a new entity.

### Component language

The syntax of the component language in Backus-Naur form is:
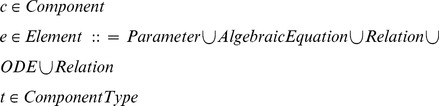


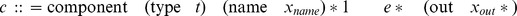


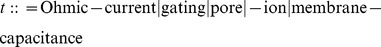



We use the set of types defined above to identify structures specific to ionic current models, although the schema of supported types can be naturally extended to support a broader range of modeling concepts. The transformation function 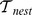 transforms the component syntax into nested environments:
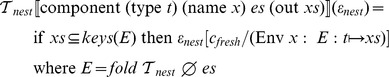


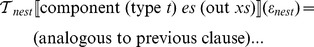


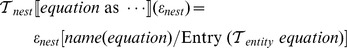



The metafunction 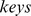 returns the set of names defined in the given environment. The transformation function 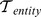 is as defined before. We do not define the case when the list of output entities is not a subset of the entities defined in the given component, but a real implementation must signal an error in such case.

Furthermore, although our definitions allow the nesting of environments, our target numerical platforms, such as Matlab, do not necessarily support namespace control. In order to generate code for such environments, we must conduct *flattening* of the nested environments, so that all identifiers can occupy the same namespace without collision.

The transformation function 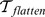 flattens nested environments by replacing all identifiers with explicit paths based on their enclosing environments:
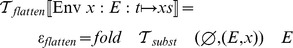


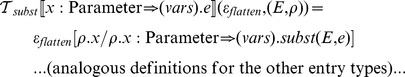


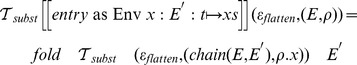



The metafunction 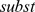 substitutes identifiers in an expression, given a substitution environment that maps identifiers to expressions. The metafunction 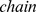 builds nested substitution environments: during the substitution process, if an identifier is not found in the immediate environment, it is looked up in the enclosing environment, and so on.

### Membrane potential transformation function

Let 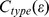 denote the set of components of type 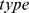 contained in the environment 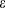. Let 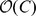 indicate the outputs declared for component 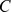. 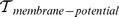 can then be defined as follows:
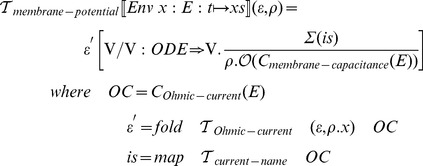


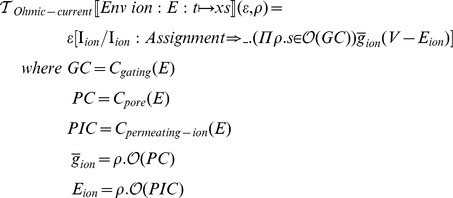


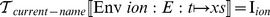




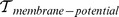
 and 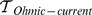 take an additional argument, 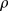, which indicates the current scope, or environment nesting path. The path specified by 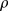 is used to disambiguate the variable names that are used in the current and voltage equations that are constructed by the transformation functions.

The transformation functions defined above require that ionic current models consist of one component of type membrane-capacitance, and one or more components of type Ohmic-current. Components of type Ohmic-current must in turn contain one or more components of type gating (gate dynamics), one component of type pore (maximal conductance) and one component of type permeating-ion (reversal potential).

The 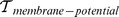 procedure takes input in the form of nested environments. We rely on the metafunctions 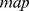 and 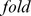 to perform operations on a list of components. 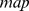 applies a given function to every member of a list of components and returns a list of the results. 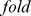 (also known as 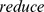 in Python or 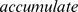 in C++) iterates a given function over a list of components and builds up a cumulative result.

### Gating dynamics transformation function

The transformation function for HH-gating-dynamics has the following definition:
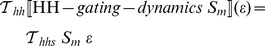


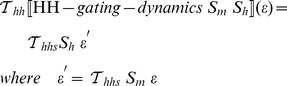


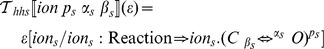


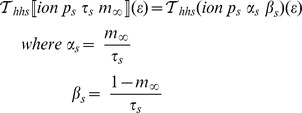



### Extended membrane potential transformation function

To support the GHK formalism, we first extend the grammar of the ionic current description language from the beginning of this section with the requisite clauses:
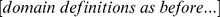


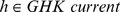


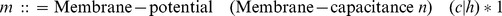


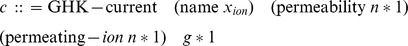


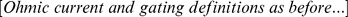
Then 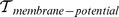 must be extended with matching clauses:
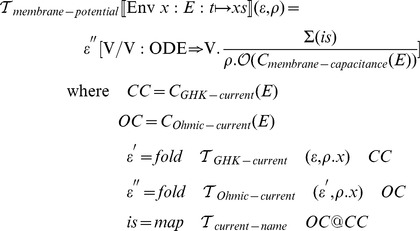


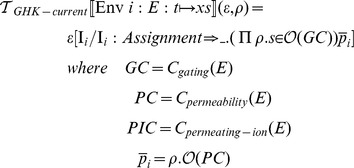



### Abstract code generation

Having defined the structures for describing ionic currents and component-based systems of equations, we can now define a transformation function that takes in an environment of entities as input and produces code for a given solver API. We abstract away the details of implementation by using idealized mathematical structures that mimic the structure of the target API. Nevertheless, we indicate what procedures are necessary to turn our abstract notation into concrete programming language syntax.

We first define a code generation function for a Matlab-like language, following the API required by the Matlab ODE solver: 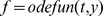 where 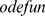 receives the independent variable 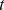 and a state vector 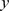 and must return the vector of derivatives that corresponds to the given input.

In order to proceed with code generation, we must have the following representation of the system of equations:

- An ordered list of parameters and algebraic assignments: if any assignments or parameters depend on one another, we must order them appropriately and ensure no circular assignments are present.
- A list of relations: relations take the form of function declarations in Matlab and most other numerical computing environments.
- A list of differential equations
- A mapping that assigns integer indices to state variable names: we use this mapping to retrieve state values from the initial state vector and to construct the vector of derivatives.

We first define transformation function, 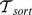, which computes the free variables of every expression and orders the entries in the environment according the dependencies in their associated expression:
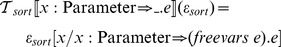


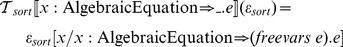


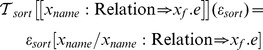


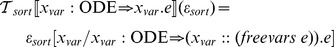


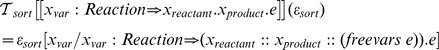
where the metafunction 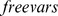 computes the free variables in an expression, and 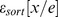 inserts a new entry in an ordered collection according to a partial order predicate:
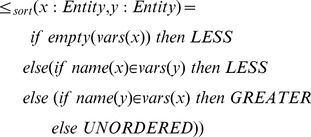



Given an ordered environment 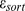, we can now define a transformation function to construct a structure suitable for input to code generation procedures. In this particular case, our target structure is a 5-tuple of the form:
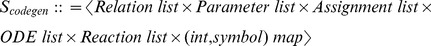
The last element in the tuple is a mapping between state vector indices and state variable names.

The transformation function can then be defined as follows:
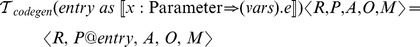


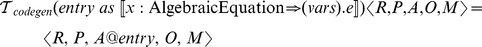


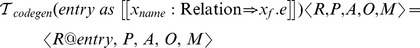


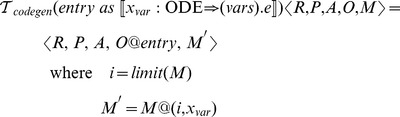


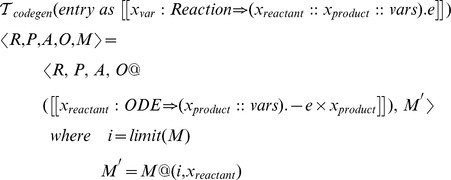
We use the 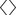 characters to indicate tuple construction, the 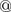 metafunction to indicate list concatenation and the 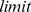 metafunction returns the largest integer plus one from the given map.

The concrete code generation procedures can be defined as follows:
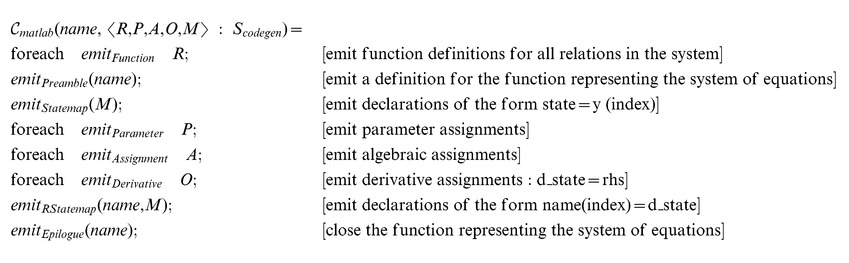
Each of the 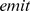 metafunctions are relatively simple procedures that map the abstract representation to the concrete syntax of the target language. foreach applies the given procedure to each element of the given list.

#### Code generation specific to neuroscience modeling software

From a code generation point of view, the component mechanism facilitates the generation of code for neuroscience-specific software environments, such as NEURON. The code generation process for NMODL uses the same transformation functions as for Matlab, but it requires one additional transformation function, 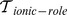.


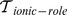
 extracts information about the ionic currents and gate complexes present in the model description and uses this information to generate declarations required for NMODL. The structure with information specific to ionic current models has the following definition:
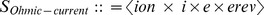


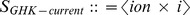


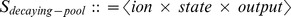


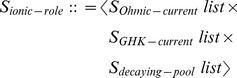



This structure contains the definitions of ionic currents, along with information about the names of permeating and accumulating ions. This is necessary in order to generate the appropriate USEION and RANGE statements for NMODL.

The transformation function to build 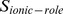 takes entities as input and can be defined as follows:
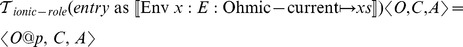


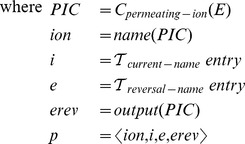


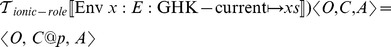


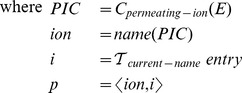


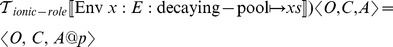


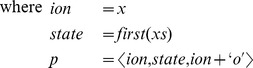
In the definition above, + is the string concatenation operator and is used to construct names for the ionic currents and reversal potentials for the specified ionic species.

The NMODL code generation function can be defined as follows:
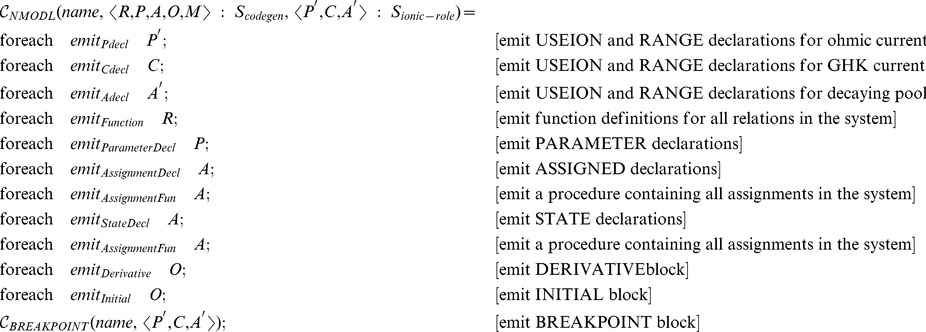


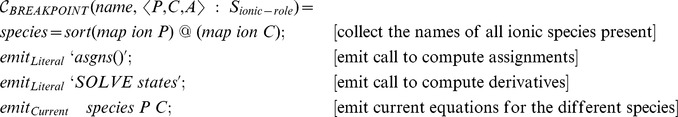



#### Simulation experiment code generation

The code generation procedures for simulation experiments are analogous with those in the previous sections. We assume that the simulation code generators for particular platforms take the form of templates instantiated by substitution, such as the ones employed by the Brian simulator [18].

In the case of voltage clamp simulation experiments, we assume the following template interface:
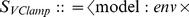


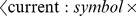


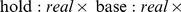


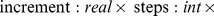


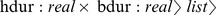
That is, we assume the target voltage clamp procedure receives a model name, names of the current variables and a list of voltage clamp parameters for each current.

The transformation function for voltage clamp script generation is then:
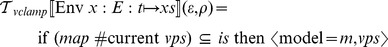


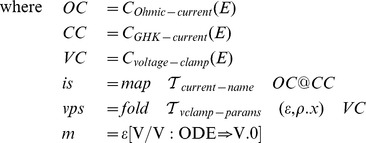


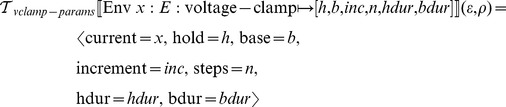
The procedure 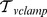 collects all voltage clamp parameter sets from the given model environment and, if the given current names correspond to the model current names, replaces the membrane potential equation with an equation that keeps the potential at the given initial value.

### Implementation study

We have conducted an implementation study of a prototype layer-oriented language for describing models of ionic currents. The implemented prototype is closely related to the semantics presented in this paper, but is not identical. The software is available for download at http://wiki.call-cc.org/nemo. It is developed in the Scheme programming language using the Chicken Scheme compiler (http://www.call-cc.org/). The Scheme and Lisp family of languages have a long tradition of domain-specific language development [48] and are intrinsically suitable for XML processing [17].

## Supporting Information

Figure S1 **NEURON simulation run times averaged over 100 trials.** A) NMODL mechanisms merged into one file; B) NMODL mechanisms in separate files. In all cases, NEURON 7.1 was used for 2000 ms of simulation time. Method cnexp indicates that NEURON's modified Crank-Nicolson method is used for solving the equations of all currents. Method cnexp+sparse indicates that NEURON's special method for kinetic equations is used for solving the equations of the resurgent sodium current and the modified Crank-Nicolson method is used for solving the equations of all other currents. Method cvode indicates that the CVODE variable step method is used for solving the equations of all currents. The hardware used was Dell Precision T5400 (CPU Intel Xeon E5430 2.66 GHz) for the Linux platform, and Apple Computer MacPro1,1 (CPU Intel Xeon 5150 2.66 GHz×2) for the Mac OS X platform.
(TIF)

Text S1 **A layer-oriented description of the 2003 Purkinje neuron model due to Khaliq et al.** Shown are two representations of the model. The first utilizes a concise parenthesized syntax, which is more convenient for human users to write. The second is canonical XML representation suitable for automatic exchange between different software. The two formats are completely interchangeable and our prototype software NEMO supports reading and writing both.
(PDF)
