# Supplementary figures and images for: The Layer-Oriented Approach to Declarative Languages for Biological Modeling
Source: PLoS Comput Biol. 2012 May 17;8(5):e1002521. doi: 10.1371/journal.pcbi.1002521 (PMC3355071; doi:10.1371/journal.pcbi.1002521)

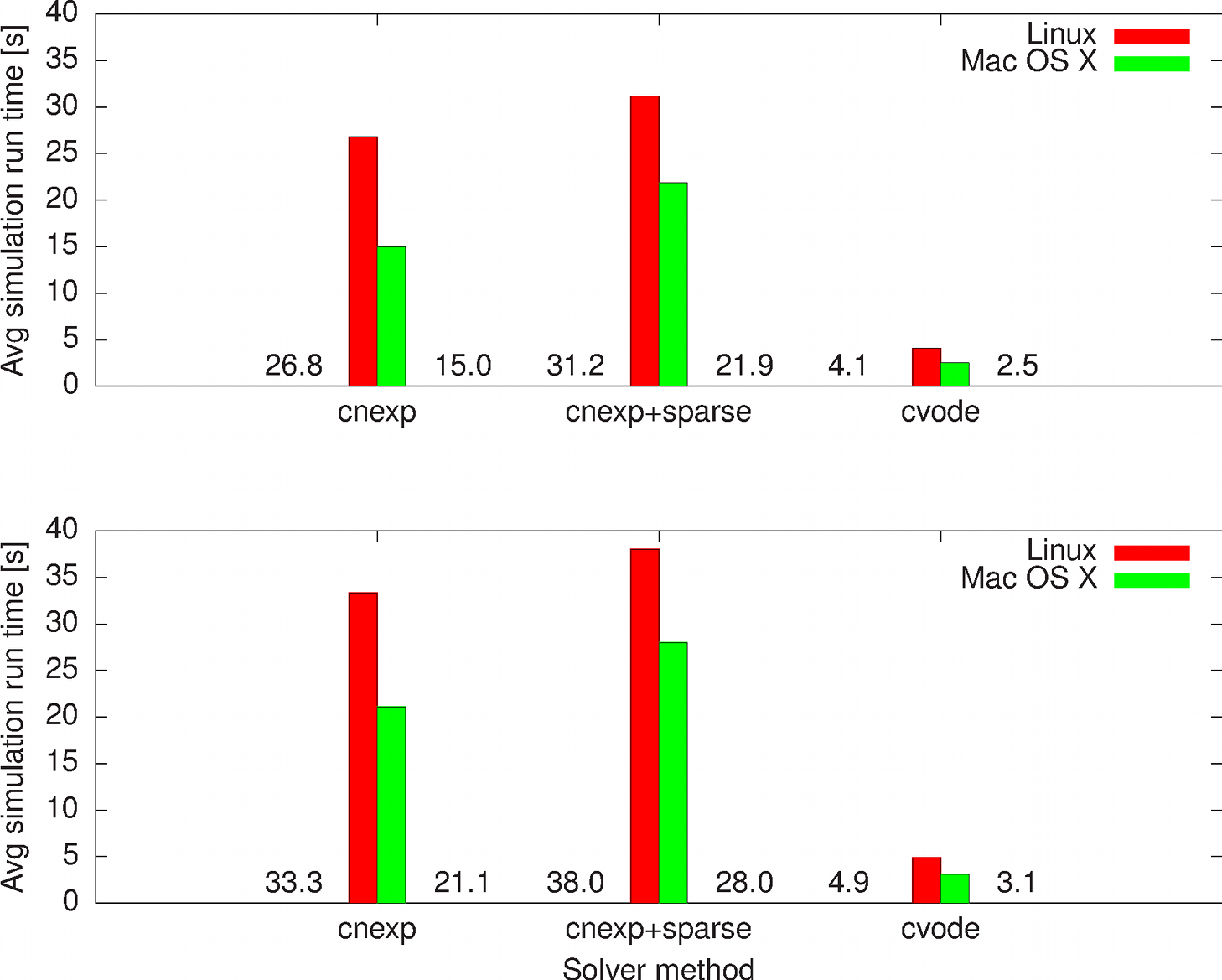

Supplement: Figure S1 — NEURON simulation run times averaged over 100 trials. A) NMODL mechanisms merged into one file; B) NMODL mechanisms in separate files. In all cases, NEURON 7.1 was used for 2000 ms of simulation time. Method cnexp indicates that NEURON's modified Crank-Nicolson method is used for solving the equations of all currents. Method cnexp+sparse indicates that NEURON's special method for kinetic equations is used for solving the equations of the resurgent sodium current and the modified Crank-Nicolson method is used for solving the equations of all other currents. Method cvode indicates that the CVODE variable step method is used for solving the equations of all currents. The hardware used was Dell Precision T5400 (CPU Intel Xeon E5430 2.66 GHz) for the Linux platform, and Apple Computer MacPro1,1 (CPU Intel Xeon 5150 2.66 GHz×2) for the Mac OS X platform. (TIF) [file pcbi.1002521.s001.tif]
