# Supplementary material for: The Layer-Oriented Approach to Declarative Languages for Biological Modeling
Source: PLoS Comput Biol. 2012 May 17;8(5):e1002521. doi: 10.1371/journal.pcbi.1002521 (PMC3355071; doi:10.1371/journal.pcbi.1002521)
Supplement: Text S1 — A layer-oriented description of the 2003 Purkinje neuron model due to Khaliq et al. Shown are two representations of the model. The first utilizes a concise parenthesized syntax, which is more convenient for human users to write. The second is canonical XML representation suitable for automatic exchange between different software. The two formats are completely interchangeable and our prototype software NEMO supports reading and writing both. (PDF) [file pcbi.1002521.s002.pdf]

Supporting Text S1

The Layer-Oriented Approach to Declarative Languages for Biological Modeling

Ivan Raikov[1,2], Erik De Schutter[1,2]
1 University of Antwerp
2 Okinawa Institute of Science and Technology

Corresponding author:
Ivan Raikov
Email: `raikov (at) oist (dot) jp`

Khaliq-Raman model listing

Below is a layer-oriented description of the 2003 Purkinje cell model due to Khaliq et al. Shown are two representations of the model. The first utilizes a concise parenthesized syntax, which is more convenient for human users to write. The second is canonical XML representation suitable for automatic exchange between different software. The two formats are completely interchangeable and our prototype software NEMO supports reading and writing both.

Parenthesized syntax

```
(nemo-model Khaliq03

  ((input v
  (cai from ion-pools)
  (ica from ion-currents))

   (const ena = 60)
   (const ek = -88)
   (const ca0 = 1e-4)

   (component (type gate-complex) (name CaBK)
   ;: BK-type Purkinje calcium-activated potassium current

      (component (type gate)

 ;; constants
 (const ztau = 1.0)

 ;; rate functions
 (CaBK_v = (v + 5))

 (minf =
      (let ((vh -28.9)
    (k  6.2))
 (1.0 / (1.0 + exp (neg ((CaBK_v - vh) / k))))))

 (mtau =
      (let
```

```
  ((y0   0.000505)
   (vh1  -33.3)
   (k1   -10.0)
   (vh2  86.4)
   (k2   10.1))
 ((1e3) * (y0 + 1 / (exp ((CaBK_v + vh1) / k1) +
                     exp ((CaBK_v + vh2) / k2))))))

 (hinf =
       (let ((y0  0.085)
     (vh  -32.0)
     (k   5.8))
 (y0 + (1 - y0) / (1 + exp ((CaBK_v - vh) / k)))))


 (htau =
       (let ((y0   0.0019)
     (vh1  -54.2)
     (k1   -12.9)
     (vh2   48.5)
     (k2    5.2))
 ((1e3) * (y0 + 1 / (exp ((CaBK_v + vh1) / k1) + exp ((CaBK_v + vh2) / k2))))))


 (zinf =
       (let ((k 0.001))
 (1 / (1 + (k / cai)))))

 (z_alpha = (zinf / ztau))
 (z_beta  = ((1 - zinf) / ztau))

 (reaction
  (z
   (transitions (<-> O C z_alpha z_beta))
   (conserve  (1 = (O + C)))
   (initial   (let ((k 0.001))
(1 / (1 + k / ca0))))
   (open O)   (power 2)))

 (output z )


 (hh-ionic-gate
  (CaBK  ;; ion name: exported variables will be of the form {ion}_{id}
   (initial-m  (minf))
   (initial-h  (hinf))
   (m-power    3)
   (h-power    1)
   (m-inf      (minf))
   (m-tau      (mtau))
   (h-inf      (hinf))
   (h-tau      (htau))))
 )
```

```
     (component (type pore)
 (const  gbar_CaBK  = 0.007)
 (output gbar_CaBK ) )

     (component (type permeating-ion) (name k)
 (const e_CaBK = ek)
 (output e_CaBK ) )

     ) ;; end BK current


   (component (type gate-complex) (name CaP)
     ;; HH P-type Calcium current

     (component (type gate)

 ;; rate functions
 (inf =
     (let ((cv  -19)  (ck  5.5))
(1.0 / (1.0 + exp (neg ((v - cv) / ck))))))

 (tau =
     ((1e3) *
      (if (v > -50)
   then (0.000191 + (0.00376 * exp (neg (((v + 41.9) / 27.8) ^ 2))))
   else (0.00026367 + (0.1278 * exp (0.10327 * v))))))

 (hh-ionic-gate
  (CaP  ;; ion name: exported variables will be of the form {ion}_{id}
   (initial-m  (inf))
   (m-power    1)
   (h-power    0)
   (m-inf      inf)
   (m-tau      taue)))

 )

     (component (type permeability)

 (defun ghk (v ci co)
   (let ((F  9.6485e4)
 (R  8.3145)
 (T  (22 + 273.19))
 (Z  2)
 (E  ((1e-3) * v)))
    (let ((k0 ((Z * (F * E)) / (R * T))))
      (let ((k1 (exp (neg(k0))))
    (k2 (((Z ^ 2) * (E * (F ^ 2))) / (R * T))))
 (1e-6) * (if (abs (1 - k1) < 1e-6)
     then (Z * F * (ci - (co * k1)) * (1 - k0))
     else (k2 * (ci - (co * k1)) / (1 - k1))))))

 (const pcabar  = 0.00005)
 (const cao     = 2.4)
```

```
(pca           = (pcabar * ghk (v cai cao)))

(output pca ))

     (component (type permeating-ion) (name ca) )


     ) ;; end CaP current

  (component (type gate-complex) (name K1)
  ;; HH TEA-sensitive Purkinje potassium current

     (component (type gate)

;; constants

;; rate functions

(K1_v = (v + 11)) ;; account for junction potential

(minf =
      (let ((mivh -24)
    (mik  15.4))
(1 / (1 + exp (neg (K1_v - mivh) / mik)))))


(mtau =
      (let ((mty0   0.00012851)
    (mtvh1  100.7)
    (mtk1   12.9)
    (mtvh2  -56.0)
    (mtk2   -23.1))
(1e3 * (if (K1_v < -35)
   then (3.0 * (3.4225e-5 + 0.00498 * exp (neg (K1_v) / -28.29)))
   else (mty0 + 1.0 / (exp ((K1_v + mtvh1) / mtk1) + exp ((K1_v + mtvh2) / mtk2)))
   ))))

(hinf =
      (let ((hiy0  0.31)
    (hiA   0.78)
    (hivh  -5.802)
    (hik   11.2))
(hiy0 + hiA / (1 + exp ((K1_v - hivh) / hik)))))


(htau =
      (1e3 * (if ( K1_v > 0 )
 then (0.0012 + 0.0023 * exp (-0.141 * K1_v))
 else (1.2202e-05 + 0.012 * exp (neg (((K1_v - (-56.3)) / 49.6) ^ 2))))))

(hh-ionic-gate
 (K1  ;; ion name: exported variables will be of the form {ion}_{id}
  (initial-m  (minf))
  (initial-h  (hinf))
```

```
  (m-power    3)
  (h-power    1)
  (m-inf      (minf))
  (m-tau      (mtau))
  (h-inf      (hinf))
  (h-tau      (htau))))

)

     (component (type pore)
(const  gbar  = 0.004)
(output gbar ))

     (component (type permeating-ion) (name k)
(const e = ek)
(output e ))

     ) ;; end K1 current

  (component (type gate-complex) (name K2)
  ;; HH Low TEA-sensitive Purkinje potassium current

     (component (type gate)

;; constants

;; rate functions

(K2_v = (v + 11)) ;; account for junction potential

(minf =
      (let ((mivh -24)
    (mik  20.4))
(1 / (1 + exp ((neg(K2_v - mivh)) / mik)))))

(mtau =
      ((1e3) * (if (K2_v < -20)
   then (0.000688 + 1 / (exp ((K2_v + 64.2) / 6.5) + exp ((K2_v - 141.5) / -34.8)))
   else (0.00016 + 0.0008 * exp (-0.0267 * K2_v)))))

(hh-ionic-gate
 (K2  ;; ion name: exported variables will be of the form {ion}_{id}
  (initial-m  (minf))
  (m-power    4)
  (h-power    0)
  (m-inf      (minf))
  (m-tau      (mtau))))

)

     (component (type pore)
(const  gbar  = 0.002)
(output gbar ))
```

```
     (component (type permeating-ion) (name k)
(const e = ek)
(output e ))

     ) ;; end K2 current


  (component (type gate-complex) (name K3)
  ;; HH slow TEA-insensitive Purkinje potassium current

     (component (type gate)

;; constants

;; rate functions

(K3_v = (v + 11)) ;; account for junction potential

(minf =
      (let ((mivh -16.5)
    (mik  18.4))
(1 / (1 + exp ((neg(K3_v - mivh)) / mik)))))

(mtau =
      ((1e3) * (0.000796 + 1.0 / (exp ((K3_v + 73.2) / 11.7) + exp ((K3_v - 306.7) / -74.2)))))

(hh-ionic-gate
 (K3  ;; ion name: exported variables will be of the form {ion}_{id}
  (initial-m  (minf))
  (m-power    4)
  (h-power    0)
  (m-inf      (minf))
  (m-tau      (mtau))))

)

     (component (type pore)
(const  gbar  =  0.004)
(output gbar ))

     (component (type permeating-ion) (name k)
(const e = ek)
(output e ))

     ) ;; end K3 current

  (component (type gate-complex) (name Narsg)

     ;; constants

     (component (type gate)

(const Con   = 0.005)
(const Coff  = 0.5)
```

```
(const Oon   = 0.75)
(const Ooff  = 0.005)

(const alfac = (pow ((Oon / Con) (1.0 / 4.0))))
(const btfac = (pow ((Ooff / Coff) (1.0 / 4.0))))

(const alpha = 150)
(const beta  = 3)
(const gamma = 150)
(const delta = 40)
(const epsilon = 1.75)
(const zeta = 0.03)
(const x1 = 20)
(const x2 = -20)
(const x3 = 1e12)
(const x4 = -1e12)
(const x5 = 1e12)
(const x6 = -25)

;; rate functions

(f01 = (4.0 * alpha * exp (v / x1)))
(f02 = (3.0 * alpha * exp (v / x1)))
(f03 = (2.0 * alpha * exp (v / x1)))
(f04 = (alpha * exp (v / x1)))
(f0O = (gamma * exp (v / x3)))
(fip = (epsilon * exp (v / x5)))
(f11 = (4.0 * alpha * alfac * exp (v / x1)))
(f12 = (3.0 * alpha * alfac * exp (v / x1)))
(f13 = (2.0 * alpha * alfac * exp (v / x1)))
(f14 = (alpha * alfac * exp (v / x1)))
(f1n = (gamma * exp (v / x3)))

(fi1 = (Con))
(fi2 = (Con * alfac))
(fi3 = (Con * alfac * alfac))
(fi4 = (Con * alfac * alfac * alfac))
(fi5 = (Con * alfac * alfac * alfac * alfac))
(fin = (Oon))

(b01 = (beta * exp (v / x2)))
(b02 = (2.0 * beta * exp (v / x2)))
(b03 = (3.0 * beta * exp (v / x2)))
(b04 = (4.0 * beta * exp (v / x2)))
(b0O = (delta * exp (v / x4)))
(bip = (zeta * exp (v / x6)))

(b11 = (beta * btfac * exp (v / x2)))
(b12 = (2.0 * beta * btfac * exp (v / x2)))
(b13 = (3.0 * beta * btfac * exp (v / x2)))
(b14 = (4.0 * beta * btfac * exp (v / x2)))
(b1n = (delta * exp (v / x4)))

(bi1 = (Coff))
```

```
(bi2 = (Coff * btfac))
(bi3 = (Coff * btfac * btfac))
(bi4 = (Coff * btfac * btfac * btfac))
(bi5 = (Coff * btfac * btfac * btfac * btfac))
(bin = (Ooff))

(reaction
 (z
  (transitions
   (<-> C1 C2 f01 b01)
   (<-> C2 C3 f02 b02)
   (<-> C3 C4 f03 b03)
   (<-> C4 C5 f04 b04)
   (<-> C5 O  f0O b0O)
   (<-> O  B  fip bip)
   (<-> O  I6 fin bin)
   (<-> C1 I1 fi1 bi1)
   (<-> C2 I2 fi2 bi2)
   (<-> C3 I3 fi3 bi3)
   (<-> C4 I4 fi4 bi4)
   (<-> C5 I5 fi5 bi5)
   (<-> I1 I2 f11 b11)
   (<-> I2 I3 f12 b12)
   (<-> I3 I4 f13 b13)
   (<-> I4 I5 f14 b14)
   (<-> I5 I6 f1n b1n)
   )

  (conserve (1 = (I1 + I2 + I3 + I4 + I5 + I6 + C1 + C2 + C3 + C4 + C5 + O + B)))

  (open O)   (power 1)))

(output z )

)

     (component (type pore)
(const  gbar  = 0.015)
(output gbar ))

     (component (type permeating-ion) (name na)
(const e = ena)
(output e ))

     ) ;; end Narsg component


  (component (type gate-complex) (name Ih)

     (component (type gate)

;; rate functions

(inf = (1.0 /(1.0 + exp ((v + 90.1) / 9.9))))
```

```
(tau = ((1e3) * (0.19 + 0.72 * exp (neg(((v - (-81.5)) / 11.9) ^ 2)))))

(hh-ionic-gate
 (Ih  ;; ion name: exported variables will be of the form {ion}_{id}
  (initial-m (inf))
  (m-power   1)
  (h-power   0)
  (m-inf     (inf))
  (m-tau     (tau))
  ))

)

     (component (type pore)
(const  gbar  = 0.0001)
(output gbar ))

     (component (type permeating-ion) (name non-specific)
(const e = -30)
(output e ))

     ) ;; end Ih current


  (component (type gate-complex) (name Leak)

     (component (type pore)
(const  gbar  = 5e-5)
(output gbar ))

     (component (type permeating-ion) (name non-specific)
(const e = -60)
(output e ))

     ) ;; end leak current


  (component (type decaying-pool) (name ca)

     (const  F = 96485.0)
     (const  ca_depth   = 0.1)
     (const  ca_beta    = 1.0)

     (d (ca) =  ((neg (ica) / (2 * ca0 * F * ca_depth)) -
 ((if (ca < ca0) then ca0 else ca) * ca_beta))
(initial ca0))

     (cac = (if (ca < ca0) then ca0 else ca))

     (output cac)
     )
```

```
   (component (type membrane-capacitance)
           (const C_m  = (1e-3))
   (output C_m))

))
```

XML syntax

```
<?xml version="1.0" ?>

<ncml:model xmlns="ncml" xmlns:ncml="ncml" name="Khaliq03">
<ncml:input id="v">
<as>v</as></ncml:input>
<ncml:input id="cai">
<as>cai</as>
<from>ion-pools</from></ncml:input>
<ncml:input id="ica">
<as>ica</as>
<from>ion-currents</from></ncml:input>
<ncml:const id="ena">
<ncml:expr>60.0</ncml:expr></ncml:const>
<ncml:const id="ek">
<ncml:expr>-88.0</ncml:expr></ncml:const>
<ncml:const id="ca0">
<ncml:expr>0.0001</ncml:expr></ncml:const>
<ncml:component name="CaBK" type="gate-complex">
<ncml:component name="comp1" type="gate">
<ncml:const id="comp0:ztau">
<ncml:expr>1.0</ncml:expr></ncml:const>
<ncml:asgn id="comp0:CaBK_v">
<ncml:expr>
<ncml:sum>
<ncml:id>v</ncml:id>5.0</ncml:sum></ncml:expr></ncml:asgn>
<ncml:asgn id="comp0:minf">
<ncml:expr>
<ncml:let>
<ncml:bnds>
<ncml:bnd id="v3">
<ncml:expr>-28.9</ncml:expr></ncml:bnd>
<ncml:bnd id="v2">
<ncml:expr>6.2</ncml:expr></ncml:bnd></ncml:bnds>
<ncml:expr>
<ncml:div>1.0
<ncml:sum>1.0
<ncml:exp>
<ncml:neg>
<ncml:div>
<ncml:sum>
<ncml:id>comp0:CaBK_v</ncml:id>
<ncml:neg>
<ncml:id>v3</ncml:id></ncml:neg></ncml:sum>
<ncml:id>v2</ncml:id></ncml:div></ncml:neg></ncml:exp></ncml:sum></ncml:div></ncml:expr></ncml:let></ncml:expr><
<ncml:asgn id="comp0:mtau">
```

```
<ncml:expr>
<ncml:let>
<ncml:bnds>
<ncml:bnd id="v8">
<ncml:expr>0.000505</ncml:expr></ncml:bnd>
<ncml:bnd id="v7">
<ncml:expr>-33.3</ncml:expr></ncml:bnd>
<ncml:bnd id="v6">
<ncml:expr>-10.0</ncml:expr></ncml:bnd>
<ncml:bnd id="v5">
<ncml:expr>86.4</ncml:expr></ncml:bnd>
<ncml:bnd id="v4">
<ncml:expr>10.1</ncml:expr></ncml:bnd></ncml:bnds>
<ncml:expr>
<ncml:mul>1000.0
<ncml:sum>
<ncml:id>v8</ncml:id>
<ncml:div>1.0
<ncml:sum>
<ncml:exp>
<ncml:div>
<ncml:sum>
<ncml:id>comp0:CaBK_v</ncml:id>
<ncml:id>v7</ncml:id></ncml:sum>
<ncml:id>v6</ncml:id></ncml:div></ncml:exp>
<ncml:exp>
<ncml:div>
<ncml:sum>
<ncml:id>comp0:CaBK_v</ncml:id>
<ncml:id>v5</ncml:id></ncml:sum>
<ncml:id>v4</ncml:id></ncml:div></ncml:exp></ncml:sum></ncml:div></ncml:sum></ncml:mul></ncml:expr></ncml:let></
<ncml:asgn id="comp0:hinf">
<ncml:expr>
<ncml:let>
<ncml:bnds>
<ncml:bnd id="v11">
<ncml:expr>0.085</ncml:expr></ncml:bnd>
<ncml:bnd id="v10">
<ncml:expr>-32.0</ncml:expr></ncml:bnd>
<ncml:bnd id="v9">
<ncml:expr>5.8</ncml:expr></ncml:bnd></ncml:bnds>
<ncml:expr>
<ncml:sum>
<ncml:id>v11</ncml:id>
<ncml:div>
<ncml:sum>1.0
<ncml:neg>
<ncml:id>v11</ncml:id></ncml:neg></ncml:sum>
<ncml:sum>1.0
<ncml:exp>
<ncml:div>
<ncml:sum>
<ncml:id>comp0:CaBK_v</ncml:id>
<ncml:neg>
```

```
<ncml:id>v10</ncml:id></ncml:neg></ncml:sum>
<ncml:id>v9</ncml:id></ncml:div></ncml:exp></ncml:sum></ncml:div></ncml:sum></ncml:expr></ncml:let></ncml:expr><
<ncml:asgn id="comp0:htau">
<ncml:expr>
<ncml:let>
<ncml:bnds>
<ncml:bnd id="v16">
<ncml:expr>0.0019</ncml:expr></ncml:bnd>
<ncml:bnd id="v15">
<ncml:expr>-54.2</ncml:expr></ncml:bnd>
<ncml:bnd id="v14">
<ncml:expr>-12.9</ncml:expr></ncml:bnd>
<ncml:bnd id="v13">
<ncml:expr>48.5</ncml:expr></ncml:bnd>
<ncml:bnd id="v12">
<ncml:expr>5.2</ncml:expr></ncml:bnd></ncml:bnds>
<ncml:expr>
<ncml:mul>1000.0
<ncml:sum>
<ncml:id>v16</ncml:id>
<ncml:div>1.0
<ncml:sum>
<ncml:exp>
<ncml:div>
<ncml:sum>
<ncml:id>comp0:CaBK_v</ncml:id>
<ncml:id>v15</ncml:id></ncml:sum>
<ncml:id>v14</ncml:id></ncml:div></ncml:exp>
<ncml:exp>
<ncml:div>
<ncml:sum>
<ncml:id>comp0:CaBK_v</ncml:id>
<ncml:id>v13</ncml:id></ncml:sum>
<ncml:id>v12</ncml:id></ncml:div></ncml:exp></ncml:sum></ncml:div></ncml:sum></ncml:mul></ncml:expr></ncml:let><
<ncml:asgn id="comp0:zinf">
<ncml:expr>
<ncml:let>
<ncml:bnds>
<ncml:bnd id="v17">
<ncml:expr>0.001</ncml:expr></ncml:bnd></ncml:bnds>
<ncml:expr>
<ncml:div>1.0
<ncml:sum>1.0
<ncml:div>
<ncml:id>v17</ncml:id>
<ncml:id>cai</ncml:id></ncml:div></ncml:sum></ncml:div></ncml:expr></ncml:let></ncml:expr></ncml:asgn>
<ncml:asgn id="comp0:z_alpha">
<ncml:expr>
<ncml:div>
<ncml:id>comp0:zinf</ncml:id>
<ncml:id>comp0:ztau</ncml:id></ncml:div></ncml:expr></ncml:asgn>
<ncml:asgn id="comp0:z_beta">
<ncml:expr>
<ncml:div>
```

```
<ncml:sum>1.0
<ncml:neg>
<ncml:id>comp0:zinf</ncml:id></ncml:neg></ncml:sum>
<ncml:id>comp0:ztau</ncml:id></ncml:div></ncml:expr></ncml:asgn>
<ncml:reaction id="comp0:z">
<ncml:open>O</ncml:open>
<ncml:initial>0.0909090909090909</ncml:initial>
<ncml:conserve>
<ncml:conseq val="1">
<ncml:expr>
<ncml:sum>
<ncml:id>O</ncml:id>
<ncml:id>C</ncml:id></ncml:sum></ncml:expr></ncml:conseq></ncml:conserve>
<ncml:transitions>
<ncml:transition src="O" dst="C">
<ncml:rate>
<ncml:id>comp0:z_alpha</ncml:id></ncml:rate></ncml:transition>
<ncml:transition src="C" dst="O">
<ncml:rate>
<ncml:id>comp0:z_beta</ncml:id></ncml:rate></ncml:transition></ncml:transitions>
<ncml:power>2.0</ncml:power></ncml:reaction>
<ncml:asgn id="CaBK_m-inf">
<ncml:expr>
<ncml:id>comp0:minf</ncml:id></ncml:expr></ncml:asgn>
<ncml:asgn id="CaBK_m-tau">
<ncml:expr>
<ncml:id>comp0:mtau</ncml:id></ncml:expr></ncml:asgn>
<ncml:reaction id="CaBK_m">
<ncml:open>O</ncml:open>
<ncml:initial>
<ncml:id>comp0:minf</ncml:id></ncml:initial>
<ncml:conserve>
<ncml:conseq val="1">
<ncml:expr>
<ncml:sum>
<ncml:id>C</ncml:id>
<ncml:id>O</ncml:id></ncml:sum></ncml:expr></ncml:conseq></ncml:conserve>
<ncml:transitions>
<ncml:transition src="C" dst="O">
<ncml:rate>
<ncml:let>
<ncml:bnds>
<ncml:bnd id="v97">
<ncml:expr>
<ncml:div>
<ncml:id>CaBK_m-inf</ncml:id>
<ncml:id>CaBK_m-tau</ncml:id></ncml:div></ncml:expr></ncml:bnd></ncml:bnds>
<ncml:expr>
<ncml:id>v97</ncml:id></ncml:expr></ncml:let></ncml:rate></ncml:transition>
<ncml:transition src="O" dst="C">
<ncml:rate>
<ncml:let>
<ncml:bnds>
<ncml:bnd id="v98">
```

```
<ncml:expr>
<ncml:div>
<ncml:sum>1.0
<ncml:neg>
<ncml:id>CaBK_m-inf</ncml:id></ncml:neg></ncml:sum>
<ncml:id>CaBK_m-tau</ncml:id></ncml:div></ncml:expr></ncml:bnd></ncml:bnds>
<ncml:expr>
<ncml:id>v98</ncml:id></ncml:expr></ncml:let></ncml:rate></ncml:transition></ncml:transitions>
<ncml:power>3.0</ncml:power></ncml:reaction>
<ncml:asgn id="CaBK_h-inf">
<ncml:expr>
<ncml:id>comp0:hinf</ncml:id></ncml:expr></ncml:asgn>
<ncml:asgn id="CaBK_h-tau">
<ncml:expr>
<ncml:id>comp0:htau</ncml:id></ncml:expr></ncml:asgn>
<ncml:reaction id="CaBK_h">
<ncml:open>O</ncml:open>
<ncml:initial>
<ncml:id>comp0:hinf</ncml:id></ncml:initial>
<ncml:conserve>
<ncml:conseq val="1">
<ncml:expr>
<ncml:sum>
<ncml:id>C</ncml:id>
<ncml:id>O</ncml:id></ncml:sum></ncml:expr></ncml:conseq></ncml:conserve>
<ncml:transitions>
<ncml:transition src="C" dst="O">
<ncml:rate>
<ncml:let>
<ncml:bnds>
<ncml:bnd id="v99">
<ncml:expr>
<ncml:div>
<ncml:id>CaBK_h-inf</ncml:id>
<ncml:id>CaBK_h-tau</ncml:id></ncml:div></ncml:expr></ncml:bnd></ncml:bnds>
<ncml:expr>
<ncml:id>v99</ncml:id></ncml:expr></ncml:let></ncml:rate></ncml:transition>
<ncml:transition src="O" dst="C">
<ncml:rate>
<ncml:let>
<ncml:bnds>
<ncml:bnd id="v100">
<ncml:expr>
<ncml:div>
<ncml:sum>1.0
<ncml:neg>
<ncml:id>CaBK_h-inf</ncml:id></ncml:neg></ncml:sum>
<ncml:id>CaBK_h-tau</ncml:id></ncml:div></ncml:expr></ncml:bnd></ncml:bnds>
<ncml:expr>
<ncml:id>v100</ncml:id></ncml:expr></ncml:let></ncml:rate></ncml:transition></ncml:transitions>
<ncml:power>1.0</ncml:power></ncml:reaction>
<ncml:output id="comp0:z"></ncml:output>
<ncml:output id="CaBK_m"></ncml:output>
<ncml:output id="CaBK_h"></ncml:output></ncml:component>
```

```
<ncml:component name="comp20" type="pore">
<ncml:const id="comp0:gbar_CaBK">
<ncml:expr>0.007</ncml:expr></ncml:const>
<ncml:output id="comp0:gbar_CaBK"></ncml:output></ncml:component>
<ncml:component name="k" type="permeating-ion">
<ncml:const id="comp0:e_CaBK">
<ncml:expr>-88.0</ncml:expr></ncml:const>
<ncml:output id="comp0:e_CaBK"></ncml:output></ncml:component></ncml:component>
<ncml:component name="CaP" type="gate-complex">
<ncml:component name="comp23" type="gate">
<ncml:asgn id="comp22:inf">
<ncml:expr>
<ncml:let>
<ncml:bnds>
<ncml:bnd id="v25">
<ncml:expr>-19.0</ncml:expr></ncml:bnd>
<ncml:bnd id="v24">
<ncml:expr>5.5</ncml:expr></ncml:bnd></ncml:bnds>
<ncml:expr>
<ncml:div>1.0
<ncml:sum>1.0
<ncml:exp>
<ncml:neg>
<ncml:div>
<ncml:sum>
<ncml:id>v</ncml:id>
<ncml:neg>
<ncml:id>v25</ncml:id></ncml:neg></ncml:sum>
<ncml:id>v24</ncml:id></ncml:div></ncml:neg></ncml:exp></ncml:sum></ncml:div></ncml:expr></ncml:let></ncml:expr>
<ncml:asgn id="comp22:tau">
<ncml:expr>
<ncml:mul>1000.0
<ncml:apply id="if">
<ncml:gt>
<ncml:id>v</ncml:id>-50.0</ncml:gt>
<ncml:sum>0.000191
<ncml:mul>0.00376
<ncml:exp>
<ncml:neg>
<ncml:pow>
<ncml:div>
<ncml:sum>
<ncml:id>v</ncml:id>41.9</ncml:sum>27.8</ncml:div>2.0</ncml:pow></ncml:neg></ncml:exp></ncml:mul></ncml:sum>
<ncml:sum>0.00026367
<ncml:mul>0.1278
<ncml:exp>
<ncml:mul>0.10327
<ncml:id>v</ncml:id></ncml:mul></ncml:exp></ncml:mul></ncml:sum></ncml:apply></ncml:mul></ncml:expr></ncml:asgn>
<ncml:asgn id="CaP_m-inf">
<ncml:expr>
<ncml:id>comp22:inf</ncml:id></ncml:expr></ncml:asgn>
<ncml:asgn id="CaP_m-tau">
<ncml:expr>
<ncml:id>comp22:tau</ncml:id></ncml:expr></ncml:asgn>
```

```
<ncml:reaction id="CaP_m">
<ncml:open>O</ncml:open>
<ncml:initial>
<ncml:id>comp22:inf</ncml:id></ncml:initial>
<ncml:conserve>
<ncml:conseq val="1">
<ncml:expr>
<ncml:sum>
<ncml:id>C</ncml:id>
<ncml:id>O</ncml:id></ncml:sum></ncml:expr></ncml:conseq></ncml:conserve>
<ncml:transitions>
<ncml:transition src="C" dst="O">
<ncml:rate>
<ncml:let>
<ncml:bnds>
<ncml:bnd id="v101">
<ncml:expr>
<ncml:div>
<ncml:id>CaP_m-inf</ncml:id>
<ncml:id>CaP_m-tau</ncml:id></ncml:div></ncml:expr></ncml:bnd></ncml:bnds>
<ncml:expr>
<ncml:id>v101</ncml:id></ncml:expr></ncml:let></ncml:rate></ncml:transition>
<ncml:transition src="O" dst="C">
<ncml:rate>
<ncml:let>
<ncml:bnds>
<ncml:bnd id="v102">
<ncml:expr>
<ncml:div>
<ncml:sum>1.0
<ncml:neg>
<ncml:id>CaP_m-inf</ncml:id></ncml:neg></ncml:sum>
<ncml:id>CaP_m-tau</ncml:id></ncml:div></ncml:expr></ncml:bnd></ncml:bnds>
<ncml:expr>
<ncml:id>v102</ncml:id></ncml:expr></ncml:let></ncml:rate></ncml:transition></ncml:transitions>
<ncml:power>1.0</ncml:power></ncml:reaction>
<ncml:output id="CaP_m"></ncml:output></ncml:component>
<ncml:component name="comp26" type="permeability">
<ncml:defun id="comp22:ghk">
<ncml:arg>v</ncml:arg>
<ncml:arg>ci</ncml:arg>
<ncml:arg>co</ncml:arg>
<ncml:body>
<ncml:let>
<ncml:bnds>
<ncml:bnd id="v31">
<ncml:expr>96485.0</ncml:expr></ncml:bnd>
<ncml:bnd id="v30">
<ncml:expr>8.3145</ncml:expr></ncml:bnd>
<ncml:bnd id="v29">
<ncml:expr>
<ncml:sum>22.0273.19</ncml:sum></ncml:expr></ncml:bnd>
<ncml:bnd id="v28">
<ncml:expr>2.0</ncml:expr></ncml:bnd>
```

```
<ncml:bnd id="v27">
<ncml:expr>
<ncml:mul>0.001
<ncml:id>v</ncml:id></ncml:mul></ncml:expr></ncml:bnd></ncml:bnds>
<ncml:expr>
<ncml:let>
<ncml:bnds>
<ncml:bnd id="v34">
<ncml:expr>
<ncml:div>
<ncml:mul>
<ncml:id>v28</ncml:id>
<ncml:mul>
<ncml:id>v31</ncml:id>
<ncml:id>v27</ncml:id></ncml:mul></ncml:mul>
<ncml:mul>
<ncml:id>v30</ncml:id>
<ncml:id>v29</ncml:id></ncml:mul></ncml:div></ncml:expr></ncml:bnd></ncml:bnds>
<ncml:expr>
<ncml:let>
<ncml:bnds>
<ncml:bnd id="v36">
<ncml:expr>
<ncml:exp>
<ncml:neg>
<ncml:id>v34</ncml:id></ncml:neg></ncml:exp></ncml:expr></ncml:bnd>
<ncml:bnd id="v35">
<ncml:expr>
<ncml:div>
<ncml:mul>
<ncml:pow>
<ncml:id>v28</ncml:id>2.0</ncml:pow>
<ncml:mul>
<ncml:id>v27</ncml:id>
<ncml:pow>
<ncml:id>v31</ncml:id>2.0</ncml:pow></ncml:mul></ncml:mul>
<ncml:mul>
<ncml:id>v30</ncml:id>
<ncml:id>v29</ncml:id></ncml:mul></ncml:div></ncml:expr></ncml:bnd></ncml:bnds>
<ncml:expr>
<ncml:mul>1e-06
<ncml:apply id="if">
<ncml:lt>
<ncml:abs>
<ncml:sum>1.0
<ncml:neg>
<ncml:id>v36</ncml:id></ncml:neg></ncml:sum></ncml:abs>1e-06</ncml:lt>
<ncml:mul>
<ncml:mul>
<ncml:mul>
<ncml:id>v28</ncml:id>
<ncml:id>v31</ncml:id></ncml:mul>
<ncml:sum>
<ncml:id>ci</ncml:id>
```

```
<ncml:neg>
<ncml:mul>
<ncml:id>co</ncml:id>
<ncml:id>v36</ncml:id></ncml:mul></ncml:neg></ncml:sum></ncml:mul>
<ncml:sum>1.0
<ncml:neg>
<ncml:id>v34</ncml:id></ncml:neg></ncml:sum></ncml:mul>
<ncml:div>
<ncml:mul>
<ncml:id>v35</ncml:id>
<ncml:sum>
<ncml:id>ci</ncml:id>
<ncml:neg>
<ncml:mul>
<ncml:id>co</ncml:id>
<ncml:id>v36</ncml:id></ncml:mul></ncml:neg></ncml:sum></ncml:mul>
<ncml:sum>1.0
<ncml:neg>
<ncml:id>v36</ncml:id></ncml:neg></ncml:sum></ncml:div></ncml:apply></ncml:mul></ncml:expr></ncml:let></ncml:exp
<ncml:const id="comp22:pcabar">
<ncml:expr>5e-05</ncml:expr></ncml:const>
<ncml:const id="comp22:cao">
<ncml:expr>2.4</ncml:expr></ncml:const>
<ncml:asgn id="comp22:pca">
<ncml:expr>
<ncml:mul>
<ncml:id>comp22:pcabar</ncml:id>
<ncml:apply id="comp22:ghk">
<ncml:id>v</ncml:id>
<ncml:id>cai</ncml:id>
<ncml:id>comp22:cao</ncml:id></ncml:apply></ncml:mul></ncml:expr></ncml:asgn>
<ncml:output id="comp22:pca"></ncml:output></ncml:component>
<ncml:component name="ca" type="permeating-ion"></ncml:component></ncml:component>
<ncml:component name="K1" type="gate-complex">
<ncml:component name="comp58" type="gate">
<ncml:asgn id="comp57:K1_v">
<ncml:expr>
<ncml:sum>
<ncml:id>v</ncml:id>11.0</ncml:sum></ncml:expr></ncml:asgn>
<ncml:asgn id="comp57:minf">
<ncml:expr>
<ncml:let>
<ncml:bnds>
<ncml:bnd id="v60">
<ncml:expr>-24.0</ncml:expr></ncml:bnd>
<ncml:bnd id="v59">
<ncml:expr>15.4</ncml:expr></ncml:bnd></ncml:bnds>
<ncml:expr>
<ncml:div>1.0
<ncml:sum>1.0
<ncml:exp>
<ncml:div>
<ncml:neg>
<ncml:sum>
```

```
<ncml:id>comp57:K1_v</ncml:id>
<ncml:neg>
<ncml:id>v60</ncml:id></ncml:neg></ncml:sum></ncml:neg>
<ncml:id>v59</ncml:id></ncml:div></ncml:exp></ncml:sum></ncml:div></ncml:expr></ncml:let></ncml:expr></ncml:asgn
<ncml:asgn id="comp57:mtau">
<ncml:expr>
<ncml:let>
<ncml:bnds>
<ncml:bnd id="v65">
<ncml:expr>0.00012851</ncml:expr></ncml:bnd>
<ncml:bnd id="v64">
<ncml:expr>100.7</ncml:expr></ncml:bnd>
<ncml:bnd id="v63">
<ncml:expr>12.9</ncml:expr></ncml:bnd>
<ncml:bnd id="v62">
<ncml:expr>-56.0</ncml:expr></ncml:bnd>
<ncml:bnd id="v61">
<ncml:expr>-23.1</ncml:expr></ncml:bnd></ncml:bnds>
<ncml:expr>
<ncml:mul>1000.0
<ncml:apply id="if">
<ncml:lt>
<ncml:id>comp57:K1_v</ncml:id>-35.0</ncml:lt>
<ncml:mul>3.0
<ncml:sum>3.4225e-05
<ncml:mul>0.00498
<ncml:exp>
<ncml:div>
<ncml:neg>
<ncml:id>comp57:K1_v</ncml:id></ncml:neg>-28.29</ncml:div></ncml:exp></ncml:mul></ncml:sum></ncml:mul>
<ncml:sum>
<ncml:id>v65</ncml:id>
<ncml:div>1.0
<ncml:sum>
<ncml:exp>
<ncml:div>
<ncml:sum>
<ncml:id>comp57:K1_v</ncml:id>
<ncml:id>v64</ncml:id></ncml:sum>
<ncml:id>v63</ncml:id></ncml:div></ncml:exp>
<ncml:exp>
<ncml:div>
<ncml:sum>
<ncml:id>comp57:K1_v</ncml:id>
<ncml:id>v62</ncml:id></ncml:sum>
<ncml:id>v61</ncml:id></ncml:div></ncml:exp></ncml:sum></ncml:div></ncml:sum></ncml:apply></ncml:mul></ncml:expr
<ncml:asgn id="comp57:hinf">
<ncml:expr>
<ncml:let>
<ncml:bnds>
<ncml:bnd id="v69">
<ncml:expr>0.31</ncml:expr></ncml:bnd>
<ncml:bnd id="v68">
<ncml:expr>0.78</ncml:expr></ncml:bnd>
```

```
<ncml:bnd id="v67">
<ncml:expr>-5.802</ncml:expr></ncml:bnd>
<ncml:bnd id="v66">
<ncml:expr>11.2</ncml:expr></ncml:bnd></ncml:bnds>
<ncml:expr>
<ncml:sum>
<ncml:id>v69</ncml:id>
<ncml:div>
<ncml:id>v68</ncml:id>
<ncml:sum>1.0
<ncml:exp>
<ncml:div>
<ncml:sum>
<ncml:id>comp57:K1_v</ncml:id>
<ncml:neg>
<ncml:id>v67</ncml:id></ncml:neg></ncml:sum>
<ncml:id>v66</ncml:id></ncml:div></ncml:exp></ncml:sum></ncml:div></ncml:sum></ncml:expr></ncml:let></ncml:expr>
<ncml:asgn id="comp57:htau">
<ncml:expr>
<ncml:mul>1000.0
<ncml:apply id="if">
<ncml:gt>
<ncml:id>comp57:K1_v</ncml:id>0.0</ncml:gt>
<ncml:sum>0.0012
<ncml:mul>0.0023
<ncml:exp>
<ncml:mul>-0.141
<ncml:id>comp57:K1_v</ncml:id></ncml:mul></ncml:exp></ncml:mul></ncml:sum>
<ncml:sum>1.2202e-05
<ncml:mul>0.012
<ncml:exp>
<ncml:neg>
<ncml:pow>
<ncml:div>
<ncml:sum>
<ncml:id>comp57:K1_v</ncml:id>56.3</ncml:sum>49.6</ncml:div>2.0</ncml:pow></ncml:neg></ncml:exp></ncml:mul></ncm[illegible]
<ncml:asgn id="K1_m-inf">
<ncml:expr>
<ncml:id>comp57:minf</ncml:id></ncml:expr></ncml:asgn>
<ncml:asgn id="K1_m-tau">
<ncml:expr>
<ncml:id>comp57:mtau</ncml:id></ncml:expr></ncml:asgn>
<ncml:reaction id="K1_m">
<ncml:open>O</ncml:open>
<ncml:initial>
<ncml:id>comp57:minf</ncml:id></ncml:initial>
<ncml:conserve>
<ncml:conseq val="1">
<ncml:expr>
<ncml:sum>
<ncml:id>C</ncml:id>
<ncml:id>O</ncml:id></ncml:sum></ncml:expr></ncml:conseq></ncml:conserve>
<ncml:transitions>
<ncml:transition src="C" dst="O">
```

```
<ncml:rate>
<ncml:let>
<ncml:bnds>
<ncml:bnd id="v103">
<ncml:expr>
<ncml:div>
<ncml:id>K1_m-inf</ncml:id>
<ncml:id>K1_m-tau</ncml:id></ncml:div></ncml:expr></ncml:bnd></ncml:bnds>
<ncml:expr>
<ncml:id>v103</ncml:id></ncml:expr></ncml:let></ncml:rate></ncml:transition>
<ncml:transition src="O" dst="C">
<ncml:rate>
<ncml:let>
<ncml:bnds>
<ncml:bnd id="v104">
<ncml:expr>
<ncml:div>
<ncml:sum>1.0
<ncml:neg>
<ncml:id>K1_m-inf</ncml:id></ncml:neg></ncml:sum>
<ncml:id>K1_m-tau</ncml:id></ncml:div></ncml:expr></ncml:bnd></ncml:bnds>
<ncml:expr>
<ncml:id>v104</ncml:id></ncml:expr></ncml:let></ncml:rate></ncml:transition></ncml:transitions>
<ncml:power>3.0</ncml:power></ncml:reaction>
<ncml:asgn id="K1_h-inf">
<ncml:expr>
<ncml:id>comp57:hinf</ncml:id></ncml:expr></ncml:asgn>
<ncml:asgn id="K1_h-tau">
<ncml:expr>
<ncml:id>comp57:htau</ncml:id></ncml:expr></ncml:asgn>
<ncml:reaction id="K1_h">
<ncml:open>O</ncml:open>
<ncml:initial>
<ncml:id>comp57:hinf</ncml:id></ncml:initial>
<ncml:conserve>
<ncml:conseq val="1">
<ncml:expr>
<ncml:sum>
<ncml:id>C</ncml:id>
<ncml:id>O</ncml:id></ncml:sum></ncml:expr></ncml:conseq></ncml:conserve>
<ncml:transitions>
<ncml:transition src="C" dst="O">
<ncml:rate>
<ncml:let>
<ncml:bnds>
<ncml:bnd id="v105">
<ncml:expr>
<ncml:div>
<ncml:id>K1_h-inf</ncml:id>
<ncml:id>K1_h-tau</ncml:id></ncml:div></ncml:expr></ncml:bnd></ncml:bnds>
<ncml:expr>
<ncml:id>v105</ncml:id></ncml:expr></ncml:let></ncml:rate></ncml:transition>
<ncml:transition src="O" dst="C">
<ncml:rate>
```

```
<ncml:let>
<ncml:bnds>
<ncml:bnd id="v106">
<ncml:expr>
<ncml:div>
<ncml:sum>1.0
<ncml:neg>
<ncml:id>K1_h-inf</ncml:id></ncml:neg></ncml:sum>
<ncml:id>K1_h-tau</ncml:id></ncml:div></ncml:expr></ncml:bnd></ncml:bnds>
<ncml:expr>
<ncml:id>v106</ncml:id></ncml:expr></ncml:let></ncml:rate></ncml:transition></ncml:transitions>
<ncml:power>1.0</ncml:power></ncml:reaction>
<ncml:output id="K1_m"></ncml:output>
<ncml:output id="K1_h"></ncml:output></ncml:component>
<ncml:component name="comp70" type="pore">
<ncml:const id="comp57:gbar">
<ncml:expr>0.004</ncml:expr></ncml:const>
<ncml:output id="comp57:gbar"></ncml:output></ncml:component>
<ncml:component name="k" type="permeating-ion">
<ncml:const id="comp57:e">
<ncml:expr>-88.0</ncml:expr></ncml:const>
<ncml:output id="comp57:e"></ncml:output></ncml:component></ncml:component>
<ncml:component name="K2" type="gate-complex">
<ncml:component name="comp73" type="gate">
<ncml:asgn id="comp72:K2_v">
<ncml:expr>
<ncml:sum>
<ncml:id>v</ncml:id>11.0</ncml:sum></ncml:expr></ncml:asgn>
<ncml:asgn id="comp72:minf">
<ncml:expr>
<ncml:let>
<ncml:bnds>
<ncml:bnd id="v75">
<ncml:expr>-24.0</ncml:expr></ncml:bnd>
<ncml:bnd id="v74">
<ncml:expr>20.4</ncml:expr></ncml:bnd></ncml:bnds>
<ncml:expr>
<ncml:div>1.0
<ncml:sum>1.0
<ncml:exp>
<ncml:div>
<ncml:neg>
<ncml:sum>
<ncml:id>comp72:K2_v</ncml:id>
<ncml:neg>
<ncml:id>v75</ncml:id></ncml:neg></ncml:sum></ncml:neg>
<ncml:id>v74</ncml:id></ncml:div></ncml:exp></ncml:sum></ncml:div></ncml:expr></ncml:let></ncml:expr></ncml:asgn
<ncml:asgn id="comp72:mtau">
<ncml:expr>
<ncml:mul>1000.0
<ncml:apply id="if">
<ncml:lt>
<ncml:id>comp72:K2_v</ncml:id>-20.0</ncml:lt>
<ncml:sum>0.000688
```

```
<ncml:div>1.0
<ncml:sum>
<ncml:exp>
<ncml:div>
<ncml:sum>
<ncml:id>comp72:K2_v</ncml:id>64.2</ncml:sum>6.5</ncml:div></ncml:exp>
<ncml:exp>
<ncml:div>
<ncml:sum>
<ncml:id>comp72:K2_v</ncml:id>-141.5</ncml:sum>-34.8</ncml:div></ncml:exp></ncml:sum></ncml:div></ncml:sum>
<ncml:sum>0.00016
<ncml:mul>0.0008
<ncml:exp>
<ncml:mul>-0.0267
<ncml:id>comp72:K2_v</ncml:id></ncml:mul></ncml:exp></ncml:mul></ncml:sum></ncml:apply></ncml:mul></ncml:expr></
<ncml:asgn id="K2_m-inf">
<ncml:expr>
<ncml:id>comp72:minf</ncml:id></ncml:expr></ncml:asgn>
<ncml:asgn id="K2_m-tau">
<ncml:expr>
<ncml:id>comp72:mtau</ncml:id></ncml:expr></ncml:asgn>
<ncml:reaction id="K2_m">
<ncml:open>O</ncml:open>
<ncml:initial>
<ncml:id>comp72:minf</ncml:id></ncml:initial>
<ncml:conserve>
<ncml:conseq val="1">
<ncml:expr>
<ncml:sum>
<ncml:id>C</ncml:id>
<ncml:id>O</ncml:id></ncml:sum></ncml:expr></ncml:conseq></ncml:conserve>
<ncml:transitions>
<ncml:transition src="C" dst="O">
<ncml:rate>
<ncml:let>
<ncml:bnds>
<ncml:bnd id="v107">
<ncml:expr>
<ncml:div>
<ncml:id>K2_m-inf</ncml:id>
<ncml:id>K2_m-tau</ncml:id></ncml:div></ncml:expr></ncml:bnd></ncml:bnds>
<ncml:expr>
<ncml:id>v107</ncml:id></ncml:expr></ncml:let></ncml:rate></ncml:transition>
<ncml:transition src="O" dst="C">
<ncml:rate>
<ncml:let>
<ncml:bnds>
<ncml:bnd id="v108">
<ncml:expr>
<ncml:div>
<ncml:sum>1.0
<ncml:neg>
<ncml:id>K2_m-inf</ncml:id></ncml:neg></ncml:sum>
<ncml:id>K2_m-tau</ncml:id></ncml:div></ncml:expr></ncml:bnd></ncml:bnds>
```

```
<ncml:expr>
<ncml:id>v108</ncml:id></ncml:expr></ncml:let></ncml:rate></ncml:transition></ncml:transitions>
<ncml:power>4.0</ncml:power></ncml:reaction>
<ncml:output id="K2_m"></ncml:output></ncml:component>
<ncml:component name="comp76" type="pore">
<ncml:const id="comp72:gbar">
<ncml:expr>0.002</ncml:expr></ncml:const>
<ncml:output id="comp72:gbar"></ncml:output></ncml:component>
<ncml:component name="k" type="permeating-ion">
<ncml:const id="comp72:e">
<ncml:expr>-88.0</ncml:expr></ncml:const>
<ncml:output id="comp72:e"></ncml:output></ncml:component></ncml:component>
<ncml:component name="K3" type="gate-complex">
<ncml:component name="comp79" type="gate">
<ncml:asgn id="comp78:K3_v">
<ncml:expr>
<ncml:sum>
<ncml:id>v</ncml:id>11.0</ncml:sum></ncml:expr></ncml:asgn>
<ncml:asgn id="comp78:minf">
<ncml:expr>
<ncml:let>
<ncml:bnds>
<ncml:bnd id="v81">
<ncml:expr>-16.5</ncml:expr></ncml:bnd>
<ncml:bnd id="v80">
<ncml:expr>18.4</ncml:expr></ncml:bnd></ncml:bnds>
<ncml:expr>
<ncml:div>1.0
<ncml:sum>1.0
<ncml:exp>
<ncml:div>
<ncml:neg>
<ncml:sum>
<ncml:id>comp78:K3_v</ncml:id>
<ncml:neg>
<ncml:id>v81</ncml:id></ncml:neg></ncml:sum></ncml:neg>
<ncml:id>v80</ncml:id></ncml:div></ncml:exp></ncml:sum></ncml:div></ncml:expr></ncml:let></ncml:expr></ncml:asgn
<ncml:asgn id="comp78:mtau">
<ncml:expr>
<ncml:mul>1000.0
<ncml:sum>0.000796
<ncml:div>1.0
<ncml:sum>
<ncml:exp>
<ncml:div>
<ncml:sum>
<ncml:id>comp78:K3_v</ncml:id>73.2</ncml:sum>11.7</ncml:div></ncml:exp>
<ncml:exp>
<ncml:div>
<ncml:sum>
<ncml:id>comp78:K3_v</ncml:id>-306.7</ncml:sum>-74.2</ncml:div></ncml:exp></ncml:sum></ncml:div></ncml:sum></ncm
<ncml:asgn id="K3_m-inf">
<ncml:expr>
<ncml:id>comp78:minf</ncml:id></ncml:expr></ncml:asgn>
```

```
<ncml:asgn id="K3_m-tau">
<ncml:expr>
<ncml:id>comp78:mtau</ncml:id></ncml:expr></ncml:asgn>
<ncml:reaction id="K3_m">
<ncml:open>O</ncml:open>
<ncml:initial>
<ncml:id>comp78:minf</ncml:id></ncml:initial>
<ncml:conserve>
<ncml:conseq val="1">
<ncml:expr>
<ncml:sum>
<ncml:id>C</ncml:id>
<ncml:id>O</ncml:id></ncml:sum></ncml:expr></ncml:conseq></ncml:conserve>
<ncml:transitions>
<ncml:transition src="C" dst="O">
<ncml:rate>
<ncml:let>
<ncml:bnds>
<ncml:bnd id="v109">
<ncml:expr>
<ncml:div>
<ncml:id>K3_m-inf</ncml:id>
<ncml:id>K3_m-tau</ncml:id></ncml:div></ncml:expr></ncml:bnd></ncml:bnds>
<ncml:expr>
<ncml:id>v109</ncml:id></ncml:expr></ncml:let></ncml:rate></ncml:transition>
<ncml:transition src="O" dst="C">
<ncml:rate>
<ncml:let>
<ncml:bnds>
<ncml:bnd id="v110">
<ncml:expr>
<ncml:div>
<ncml:sum>1.0
<ncml:neg>
<ncml:id>K3_m-inf</ncml:id></ncml:neg></ncml:sum>
<ncml:id>K3_m-tau</ncml:id></ncml:div></ncml:expr></ncml:bnd></ncml:bnds>
<ncml:expr>
<ncml:id>v110</ncml:id></ncml:expr></ncml:let></ncml:rate></ncml:transition></ncml:transitions>
<ncml:power>4.0</ncml:power></ncml:reaction>
<ncml:output id="K3_m"></ncml:output></ncml:component>
<ncml:component name="comp82" type="pore">
<ncml:const id="comp78:gbar">
<ncml:expr>0.004</ncml:expr></ncml:const>
<ncml:output id="comp78:gbar"></ncml:output></ncml:component>
<ncml:component name="k" type="permeating-ion">
<ncml:const id="comp78:e">
<ncml:expr>-88.0</ncml:expr></ncml:const>
<ncml:output id="comp78:e"></ncml:output></ncml:component></ncml:component>
<ncml:component name="Narsg" type="gate-complex">
<ncml:component name="comp85" type="gate">
<ncml:const id="comp84:Con">
<ncml:expr>0.005</ncml:expr></ncml:const>
<ncml:const id="comp84:Coff">
<ncml:expr>0.5</ncml:expr></ncml:const>
```

```
<ncml:const id="comp84:Oon">
<ncml:expr>0.75</ncml:expr></ncml:const>
<ncml:const id="comp84:Ooff">
<ncml:expr>0.005</ncml:expr></ncml:const>
<ncml:const id="comp84:alfac">
<ncml:expr>3.49963551158058</ncml:expr></ncml:const>
<ncml:const id="comp84:btfac">
<ncml:expr>0.316227766016838</ncml:expr></ncml:const>
<ncml:const id="comp84:alpha">
<ncml:expr>150.0</ncml:expr></ncml:const>
<ncml:const id="comp84:beta">
<ncml:expr>3.0</ncml:expr></ncml:const>
<ncml:const id="comp84:gamma">
<ncml:expr>150.0</ncml:expr></ncml:const>
<ncml:const id="comp84:delta">
<ncml:expr>40.0</ncml:expr></ncml:const>
<ncml:const id="comp84:epsilon">
<ncml:expr>1.75</ncml:expr></ncml:const>
<ncml:const id="comp84:zeta">
<ncml:expr>0.03</ncml:expr></ncml:const>
<ncml:const id="comp84:x1">
<ncml:expr>20.0</ncml:expr></ncml:const>
<ncml:const id="comp84:x2">
<ncml:expr>-20.0</ncml:expr></ncml:const>
<ncml:const id="comp84:x3">
<ncml:expr>1000000000000.0</ncml:expr></ncml:const>
<ncml:const id="comp84:x4">
<ncml:expr>-1000000000000.0</ncml:expr></ncml:const>
<ncml:const id="comp84:x5">
<ncml:expr>1000000000000.0</ncml:expr></ncml:const>
<ncml:const id="comp84:x6">
<ncml:expr>-25.0</ncml:expr></ncml:const>
<ncml:asgn id="comp84:f01">
<ncml:expr>
<ncml:mul>
<ncml:mul>4.0
<ncml:id>comp84:alpha</ncml:id></ncml:mul>
<ncml:exp>
<ncml:div>
<ncml:id>v</ncml:id>
<ncml:id>comp84:x1</ncml:id></ncml:div></ncml:exp></ncml:mul></ncml:expr></ncml:asgn>
<ncml:asgn id="comp84:f02">
<ncml:expr>
<ncml:mul>
<ncml:mul>3.0
<ncml:id>comp84:alpha</ncml:id></ncml:mul>
<ncml:exp>
<ncml:div>
<ncml:id>v</ncml:id>
<ncml:id>comp84:x1</ncml:id></ncml:div></ncml:exp></ncml:mul></ncml:expr></ncml:asgn>
<ncml:asgn id="comp84:f03">
<ncml:expr>
<ncml:mul>
<ncml:mul>2.0
```

```
<ncml:id>comp84:alpha</ncml:id></ncml:mul>
<ncml:exp>
<ncml:div>
<ncml:id>v</ncml:id>
<ncml:id>comp84:x1</ncml:id></ncml:div></ncml:exp></ncml:mul></ncml:expr></ncml:asgn>
<ncml:asgn id="comp84:f04">
<ncml:expr>
<ncml:mul>
<ncml:id>comp84:alpha</ncml:id>
<ncml:exp>
<ncml:div>
<ncml:id>v</ncml:id>
<ncml:id>comp84:x1</ncml:id></ncml:div></ncml:exp></ncml:mul></ncml:expr></ncml:asgn>
<ncml:asgn id="comp84:f00">
<ncml:expr>
<ncml:mul>
<ncml:id>comp84:gamma</ncml:id>
<ncml:exp>
<ncml:div>
<ncml:id>v</ncml:id>
<ncml:id>comp84:x3</ncml:id></ncml:div></ncml:exp></ncml:mul></ncml:expr></ncml:asgn>
<ncml:asgn id="comp84:fip">
<ncml:expr>
<ncml:mul>
<ncml:id>comp84:epsilon</ncml:id>
<ncml:exp>
<ncml:div>
<ncml:id>v</ncml:id>
<ncml:id>comp84:x5</ncml:id></ncml:div></ncml:exp></ncml:mul></ncml:expr></ncml:asgn>
<ncml:asgn id="comp84:f11">
<ncml:expr>
<ncml:mul>
<ncml:mul>
<ncml:mul>4.0
<ncml:id>comp84:alpha</ncml:id></ncml:mul>
<ncml:id>comp84:alfac</ncml:id></ncml:mul>
<ncml:exp>
<ncml:div>
<ncml:id>v</ncml:id>
<ncml:id>comp84:x1</ncml:id></ncml:div></ncml:exp></ncml:mul></ncml:expr></ncml:asgn>
<ncml:asgn id="comp84:f12">
<ncml:expr>
<ncml:mul>
<ncml:mul>
<ncml:mul>3.0
<ncml:id>comp84:alpha</ncml:id></ncml:mul>
<ncml:id>comp84:alfac</ncml:id></ncml:mul>
<ncml:exp>
<ncml:div>
<ncml:id>v</ncml:id>
<ncml:id>comp84:x1</ncml:id></ncml:div></ncml:exp></ncml:mul></ncml:expr></ncml:asgn>
<ncml:asgn id="comp84:f13">
<ncml:expr>
<ncml:mul>
```

```
<ncml:mul>
<ncml:mul>2.0
<ncml:id>comp84:alpha</ncml:id></ncml:mul>
<ncml:id>comp84:alfac</ncml:id></ncml:mul>
<ncml:exp>
<ncml:div>
<ncml:id>v</ncml:id>
<ncml:id>comp84:x1</ncml:id></ncml:div></ncml:exp></ncml:mul></ncml:expr></ncml:asgn>
<ncml:asgn id="comp84:f14">
<ncml:expr>
<ncml:mul>
<ncml:mul>
<ncml:id>comp84:alpha</ncml:id>
<ncml:id>comp84:alfac</ncml:id></ncml:mul>
<ncml:exp>
<ncml:div>
<ncml:id>v</ncml:id>
<ncml:id>comp84:x1</ncml:id></ncml:div></ncml:exp></ncml:mul></ncml:expr></ncml:asgn>
<ncml:asgn id="comp84:f1n">
<ncml:expr>
<ncml:mul>
<ncml:id>comp84:gamma</ncml:id>
<ncml:exp>
<ncml:div>
<ncml:id>v</ncml:id>
<ncml:id>comp84:x3</ncml:id></ncml:div></ncml:exp></ncml:mul></ncml:expr></ncml:asgn>
<ncml:asgn id="comp84:fi1">
<ncml:expr>
<ncml:id>comp84:Con</ncml:id></ncml:expr></ncml:asgn>
<ncml:asgn id="comp84:fi2">
<ncml:expr>
<ncml:mul>
<ncml:id>comp84:Con</ncml:id>
<ncml:id>comp84:alfac</ncml:id></ncml:mul></ncml:expr></ncml:asgn>
<ncml:asgn id="comp84:fi3">
<ncml:expr>
<ncml:mul>
<ncml:mul>
<ncml:id>comp84:Con</ncml:id>
<ncml:id>comp84:alfac</ncml:id></ncml:mul>
<ncml:id>comp84:alfac</ncml:id></ncml:mul></ncml:expr></ncml:asgn>
<ncml:asgn id="comp84:fi4">
<ncml:expr>
<ncml:mul>
<ncml:mul>
<ncml:mul>
<ncml:id>comp84:Con</ncml:id>
<ncml:id>comp84:alfac</ncml:id></ncml:mul>
<ncml:id>comp84:alfac</ncml:id></ncml:mul>
<ncml:id>comp84:alfac</ncml:id></ncml:mul></ncml:expr></ncml:asgn>
<ncml:asgn id="comp84:fi5">
<ncml:expr>
<ncml:mul>
<ncml:mul>
```

```
<ncml:mul>
<ncml:mul>
<ncml:id>comp84:Con</ncml:id>
<ncml:id>comp84:alfac</ncml:id></ncml:mul>
<ncml:id>comp84:alfac</ncml:id></ncml:mul>
<ncml:id>comp84:alfac</ncml:id></ncml:mul>
<ncml:id>comp84:alfac</ncml:id></ncml:mul></ncml:expr></ncml:asgn>
<ncml:asgn id="comp84:fin">
<ncml:expr>
<ncml:id>comp84:Oon</ncml:id></ncml:expr></ncml:asgn>
<ncml:asgn id="comp84:b01">
<ncml:expr>
<ncml:mul>
<ncml:id>comp84:beta</ncml:id>
<ncml:exp>
<ncml:div>
<ncml:id>v</ncml:id>
<ncml:id>comp84:x2</ncml:id></ncml:div></ncml:exp></ncml:mul></ncml:expr></ncml:asgn>
<ncml:asgn id="comp84:b02">
<ncml:expr>
<ncml:mul>
<ncml:mul>2.0
<ncml:id>comp84:beta</ncml:id></ncml:mul>
<ncml:exp>
<ncml:div>
<ncml:id>v</ncml:id>
<ncml:id>comp84:x2</ncml:id></ncml:div></ncml:exp></ncml:mul></ncml:expr></ncml:asgn>
<ncml:asgn id="comp84:b03">
<ncml:expr>
<ncml:mul>
<ncml:mul>3.0
<ncml:id>comp84:beta</ncml:id></ncml:mul>
<ncml:exp>
<ncml:div>
<ncml:id>v</ncml:id>
<ncml:id>comp84:x2</ncml:id></ncml:div></ncml:exp></ncml:mul></ncml:expr></ncml:asgn>
<ncml:asgn id="comp84:b04">
<ncml:expr>
<ncml:mul>
<ncml:mul>4.0
<ncml:id>comp84:beta</ncml:id></ncml:mul>
<ncml:exp>
<ncml:div>
<ncml:id>v</ncml:id>
<ncml:id>comp84:x2</ncml:id></ncml:div></ncml:exp></ncml:mul></ncml:expr></ncml:asgn>
<ncml:asgn id="comp84:b0O">
<ncml:expr>
<ncml:mul>
<ncml:id>comp84:delta</ncml:id>
<ncml:exp>
<ncml:div>
<ncml:id>v</ncml:id>
<ncml:id>comp84:x4</ncml:id></ncml:div></ncml:exp></ncml:mul></ncml:expr></ncml:asgn>
<ncml:asgn id="comp84:bip">
```

```
<ncml:expr>
<ncml:mul>
<ncml:id>comp84:zeta</ncml:id>
<ncml:exp>
<ncml:div>
<ncml:id>v</ncml:id>
<ncml:id>comp84:x6</ncml:id></ncml:div></ncml:exp></ncml:mul></ncml:expr></ncml:asgn>
<ncml:asgn id="comp84:b11">
<ncml:expr>
<ncml:mul>
<ncml:mul>
<ncml:id>comp84:beta</ncml:id>
<ncml:id>comp84:btfac</ncml:id></ncml:mul>
<ncml:exp>
<ncml:div>
<ncml:id>v</ncml:id>
<ncml:id>comp84:x2</ncml:id></ncml:div></ncml:exp></ncml:mul></ncml:expr></ncml:asgn>
<ncml:asgn id="comp84:b12">
<ncml:expr>
<ncml:mul>
<ncml:mul>
<ncml:mul>2.0
<ncml:id>comp84:beta</ncml:id></ncml:mul>
<ncml:id>comp84:btfac</ncml:id></ncml:mul>
<ncml:exp>
<ncml:div>
<ncml:id>v</ncml:id>
<ncml:id>comp84:x2</ncml:id></ncml:div></ncml:exp></ncml:mul></ncml:expr></ncml:asgn>
<ncml:asgn id="comp84:b13">
<ncml:expr>
<ncml:mul>
<ncml:mul>
<ncml:mul>3.0
<ncml:id>comp84:beta</ncml:id></ncml:mul>
<ncml:id>comp84:btfac</ncml:id></ncml:mul>
<ncml:exp>
<ncml:div>
<ncml:id>v</ncml:id>
<ncml:id>comp84:x2</ncml:id></ncml:div></ncml:exp></ncml:mul></ncml:expr></ncml:asgn>
<ncml:asgn id="comp84:b14">
<ncml:expr>
<ncml:mul>
<ncml:mul>
<ncml:mul>4.0
<ncml:id>comp84:beta</ncml:id></ncml:mul>
<ncml:id>comp84:btfac</ncml:id></ncml:mul>
<ncml:exp>
<ncml:div>
<ncml:id>v</ncml:id>
<ncml:id>comp84:x2</ncml:id></ncml:div></ncml:exp></ncml:mul></ncml:expr></ncml:asgn>
<ncml:asgn id="comp84:b1n">
<ncml:expr>
<ncml:mul>
<ncml:id>comp84:delta</ncml:id>
```

```
<ncml:exp>
<ncml:div>
<ncml:id>v</ncml:id>
<ncml:id>comp84:x4</ncml:id></ncml:div></ncml:exp></ncml:mul></ncml:expr></ncml:asgn>
<ncml:asgn id="comp84:bi1">
<ncml:expr>
<ncml:id>comp84:Coff</ncml:id></ncml:expr></ncml:asgn>
<ncml:asgn id="comp84:bi2">
<ncml:expr>
<ncml:mul>
<ncml:id>comp84:Coff</ncml:id>
<ncml:id>comp84:btfac</ncml:id></ncml:mul></ncml:expr></ncml:asgn>
<ncml:asgn id="comp84:bi3">
<ncml:expr>
<ncml:mul>
<ncml:mul>
<ncml:id>comp84:Coff</ncml:id>
<ncml:id>comp84:btfac</ncml:id></ncml:mul>
<ncml:id>comp84:btfac</ncml:id></ncml:mul></ncml:expr></ncml:asgn>
<ncml:asgn id="comp84:bi4">
<ncml:expr>
<ncml:mul>
<ncml:mul>
<ncml:mul>
<ncml:id>comp84:Coff</ncml:id>
<ncml:id>comp84:btfac</ncml:id></ncml:mul>
<ncml:id>comp84:btfac</ncml:id></ncml:mul>
<ncml:id>comp84:btfac</ncml:id></ncml:mul></ncml:expr></ncml:asgn>
<ncml:asgn id="comp84:bi5">
<ncml:expr>
<ncml:mul>
<ncml:mul>
<ncml:mul>
<ncml:mul>
<ncml:id>comp84:Coff</ncml:id>
<ncml:id>comp84:btfac</ncml:id></ncml:mul>
<ncml:id>comp84:btfac</ncml:id></ncml:mul>
<ncml:id>comp84:btfac</ncml:id></ncml:mul>
<ncml:id>comp84:btfac</ncml:id></ncml:mul></ncml:expr></ncml:asgn>
<ncml:asgn id="comp84:bin">
<ncml:expr>
<ncml:id>comp84:0off</ncml:id></ncml:expr></ncml:asgn>
<ncml:reaction id="comp84:z">
<ncml:open>0</ncml:open>
<ncml:conserve>
<ncml:conseq val="1">
<ncml:expr>
<ncml:sum>
<ncml:sum>
<ncml:sum>
<ncml:sum>
<ncml:sum>
<ncml:sum>
<ncml:sum>
```

```
<ncml:sum>
<ncml:sum>
<ncml:sum>
<ncml:sum>
<ncml:sum>
<ncml:id>I1</ncml:id>
<ncml:id>I2</ncml:id></ncml:sum>
<ncml:id>I3</ncml:id></ncml:sum>
<ncml:id>I4</ncml:id></ncml:sum>
<ncml:id>I5</ncml:id></ncml:sum>
<ncml:id>I6</ncml:id></ncml:sum>
<ncml:id>C1</ncml:id></ncml:sum>
<ncml:id>C2</ncml:id></ncml:sum>
<ncml:id>C3</ncml:id></ncml:sum>
<ncml:id>C4</ncml:id></ncml:sum>
<ncml:id>C5</ncml:id></ncml:sum>
<ncml:id>O</ncml:id></ncml:sum>
<ncml:id>B</ncml:id></ncml:sum></ncml:expr></ncml:conseq></ncml:conserve>
<ncml:transitions>
<ncml:transition src="C1" dst="C2">
<ncml:rate>
<ncml:id>comp84:f01</ncml:id></ncml:rate></ncml:transition>
<ncml:transition src="C2" dst="C1">
<ncml:rate>
<ncml:id>comp84:b01</ncml:id></ncml:rate></ncml:transition>
<ncml:transition src="C2" dst="C3">
<ncml:rate>
<ncml:id>comp84:f02</ncml:id></ncml:rate></ncml:transition>
<ncml:transition src="C3" dst="C2">
<ncml:rate>
<ncml:id>comp84:b02</ncml:id></ncml:rate></ncml:transition>
<ncml:transition src="C3" dst="C4">
<ncml:rate>
<ncml:id>comp84:f03</ncml:id></ncml:rate></ncml:transition>
<ncml:transition src="C4" dst="C3">
<ncml:rate>
<ncml:id>comp84:b03</ncml:id></ncml:rate></ncml:transition>
<ncml:transition src="C4" dst="C5">
<ncml:rate>
<ncml:id>comp84:f04</ncml:id></ncml:rate></ncml:transition>
<ncml:transition src="C5" dst="C4">
<ncml:rate>
<ncml:id>comp84:b04</ncml:id></ncml:rate></ncml:transition>
<ncml:transition src="C5" dst="O">
<ncml:rate>
<ncml:id>comp84:f0O</ncml:id></ncml:rate></ncml:transition>
<ncml:transition src="O" dst="C5">
<ncml:rate>
<ncml:id>comp84:b0O</ncml:id></ncml:rate></ncml:transition>
<ncml:transition src="O" dst="B">
<ncml:rate>
<ncml:id>comp84:fip</ncml:id></ncml:rate></ncml:transition>
<ncml:transition src="B" dst="O">
<ncml:rate>
```

```
<ncml:id>comp84:bip</ncml:id></ncml:rate></ncml:transition>
<ncml:transition src="O" dst="I6">
<ncml:rate>
<ncml:id>comp84:fin</ncml:id></ncml:rate></ncml:transition>
<ncml:transition src="I6" dst="O">
<ncml:rate>
<ncml:id>comp84:bin</ncml:id></ncml:rate></ncml:transition>
<ncml:transition src="C1" dst="I1">
<ncml:rate>
<ncml:id>comp84:fi1</ncml:id></ncml:rate></ncml:transition>
<ncml:transition src="I1" dst="C1">
<ncml:rate>
<ncml:id>comp84:bi1</ncml:id></ncml:rate></ncml:transition>
<ncml:transition src="C2" dst="I2">
<ncml:rate>
<ncml:id>comp84:fi2</ncml:id></ncml:rate></ncml:transition>
<ncml:transition src="I2" dst="C2">
<ncml:rate>
<ncml:id>comp84:bi2</ncml:id></ncml:rate></ncml:transition>
<ncml:transition src="C3" dst="I3">
<ncml:rate>
<ncml:id>comp84:fi3</ncml:id></ncml:rate></ncml:transition>
<ncml:transition src="I3" dst="C3">
<ncml:rate>
<ncml:id>comp84:bi3</ncml:id></ncml:rate></ncml:transition>
<ncml:transition src="C4" dst="I4">
<ncml:rate>
<ncml:id>comp84:fi4</ncml:id></ncml:rate></ncml:transition>
<ncml:transition src="I4" dst="C4">
<ncml:rate>
<ncml:id>comp84:bi4</ncml:id></ncml:rate></ncml:transition>
<ncml:transition src="C5" dst="I5">
<ncml:rate>
<ncml:id>comp84:fi5</ncml:id></ncml:rate></ncml:transition>
<ncml:transition src="I5" dst="C5">
<ncml:rate>
<ncml:id>comp84:bi5</ncml:id></ncml:rate></ncml:transition>
<ncml:transition src="I1" dst="I2">
<ncml:rate>
<ncml:id>comp84:f11</ncml:id></ncml:rate></ncml:transition>
<ncml:transition src="I2" dst="I1">
<ncml:rate>
<ncml:id>comp84:b11</ncml:id></ncml:rate></ncml:transition>
<ncml:transition src="I2" dst="I3">
<ncml:rate>
<ncml:id>comp84:f12</ncml:id></ncml:rate></ncml:transition>
<ncml:transition src="I3" dst="I2">
<ncml:rate>
<ncml:id>comp84:b12</ncml:id></ncml:rate></ncml:transition>
<ncml:transition src="I3" dst="I4">
<ncml:rate>
<ncml:id>comp84:f13</ncml:id></ncml:rate></ncml:transition>
<ncml:transition src="I4" dst="I3">
<ncml:rate>
```

```
<ncml:id>comp84:b13</ncml:id></ncml:rate></ncml:transition>
<ncml:transition src="I4" dst="I5">
<ncml:rate>
<ncml:id>comp84:f14</ncml:id></ncml:rate></ncml:transition>
<ncml:transition src="I5" dst="I4">
<ncml:rate>
<ncml:id>comp84:b14</ncml:id></ncml:rate></ncml:transition>
<ncml:transition src="I5" dst="I6">
<ncml:rate>
<ncml:id>comp84:f1n</ncml:id></ncml:rate></ncml:transition>
<ncml:transition src="I6" dst="I5">
<ncml:rate>
<ncml:id>comp84:b1n</ncml:id></ncml:rate></ncml:transition></ncml:transitions>
<ncml:power>1.0</ncml:power></ncml:reaction>
<ncml:output id="comp84:z"></ncml:output></ncml:component>
<ncml:component name="comp86" type="pore">
<ncml:const id="comp84:gbar">
<ncml:expr>0.015</ncml:expr></ncml:const>
<ncml:output id="comp84:gbar"></ncml:output></ncml:component>
<ncml:component name="na" type="permeating-ion">
<ncml:const id="comp84:e">
<ncml:expr>60.0</ncml:expr></ncml:const>
<ncml:output id="comp84:e"></ncml:output></ncml:component></ncml:component>
<ncml:component name="Ih" type="gate-complex">
<ncml:component name="comp89" type="gate">
<ncml:asgn id="comp88:inf">
<ncml:expr>
<ncml:div>1.0
<ncml:sum>1.0
<ncml:exp>
<ncml:div>
<ncml:sum>
<ncml:id>v</ncml:id>90.1</ncml:sum>9.9</ncml:div></ncml:exp></ncml:sum></ncml:div></ncml:expr></ncml:asgn>
<ncml:asgn id="comp88:tau">
<ncml:expr>
<ncml:mul>1000.0
<ncml:sum>0.19
<ncml:mul>0.72
<ncml:exp>
<ncml:neg>
<ncml:pow>
<ncml:div>
<ncml:sum>
<ncml:id>v</ncml:id>81.5</ncml:sum>11.9</ncml:div>2.0</ncml:pow></ncml:neg></ncml:exp></ncml:mul></ncml:sum></nc
<ncml:asgn id="Ih_m-inf">
<ncml:expr>
<ncml:id>comp88:inf</ncml:id></ncml:expr></ncml:asgn>
<ncml:asgn id="Ih_m-tau">
<ncml:expr>
<ncml:id>comp88:tau</ncml:id></ncml:expr></ncml:asgn>
<ncml:reaction id="Ih_m">
<ncml:open>O</ncml:open>
<ncml:initial>
<ncml:id>comp88:inf</ncml:id></ncml:initial>
```

```
<ncml:conserve>
<ncml:conseq val="1">
<ncml:expr>
<ncml:sum>
<ncml:id>C</ncml:id>
<ncml:id>O</ncml:id></ncml:sum></ncml:expr></ncml:conseq></ncml:conserve>
<ncml:transitions>
<ncml:transition src="C" dst="O">
<ncml:rate>
<ncml:let>
<ncml:bnds>
<ncml:bnd id="v111">
<ncml:expr>
<ncml:div>
<ncml:id>Ih_m-inf</ncml:id>
<ncml:id>Ih_m-tau</ncml:id></ncml:div></ncml:expr></ncml:bnd></ncml:bnds>
<ncml:expr>
<ncml:id>v111</ncml:id></ncml:expr></ncml:let></ncml:rate></ncml:transition>
<ncml:transition src="O" dst="C">
<ncml:rate>
<ncml:let>
<ncml:bnds>
<ncml:bnd id="v112">
<ncml:expr>
<ncml:div>
<ncml:sum>1.0
<ncml:neg>
<ncml:id>Ih_m-inf</ncml:id></ncml:neg></ncml:sum>
<ncml:id>Ih_m-tau</ncml:id></ncml:div></ncml:expr></ncml:bnd></ncml:bnds>
<ncml:expr>
<ncml:id>v112</ncml:id></ncml:expr></ncml:let></ncml:rate></ncml:transition></ncml:transitions>
<ncml:power>1.0</ncml:power></ncml:reaction>
<ncml:output id="Ih_m"></ncml:output></ncml:component>
<ncml:component name="comp90" type="pore">
<ncml:const id="comp88:gbar">
<ncml:expr>0.0001</ncml:expr></ncml:const>
<ncml:output id="comp88:gbar"></ncml:output></ncml:component>
<ncml:component name="non-specific" type="permeating-ion">
<ncml:const id="comp88:e">
<ncml:expr>-30.0</ncml:expr></ncml:const>
<ncml:output id="comp88:e"></ncml:output></ncml:component></ncml:component>
<ncml:component name="Leak" type="gate-complex">
<ncml:component name="comp93" type="pore">
<ncml:const id="comp92:gbar">
<ncml:expr>5e-05</ncml:expr></ncml:const>
<ncml:output id="comp92:gbar"></ncml:output></ncml:component>
<ncml:component name="non-specific" type="permeating-ion">
<ncml:const id="comp92:e">
<ncml:expr>-60.0</ncml:expr></ncml:const>
<ncml:output id="comp92:e"></ncml:output></ncml:component></ncml:component>
<ncml:component name="ca" type="decaying-pool">
<ncml:const id="comp95:F">
<ncml:expr>96485.0</ncml:expr></ncml:const>
<ncml:const id="comp95:ca_depth">
```

```
<ncml:expr>0.1</ncml:expr></ncml:const>
<ncml:const id="comp95:ca_beta">
<ncml:expr>1.0</ncml:expr></ncml:const>
<ncml:rate id="comp95:ca">
<ncml:initial>0.0001</ncml:initial>
<ncml:expr>
<ncml:sum>
<ncml:div>
<ncml:neg>
<ncml:id>ica</ncml:id></ncml:neg>
<ncml:mul>
<ncml:mul>
<ncml:mul>2.0
<ncml:id>ca0</ncml:id></ncml:mul>
<ncml:id>comp95:F</ncml:id></ncml:mul>
<ncml:id>comp95:ca_depth</ncml:id></ncml:mul></ncml:div>
<ncml:neg>
<ncml:mul>
<ncml:apply id="if">
<ncml:lt>
<ncml:id>comp95:ca</ncml:id>
<ncml:id>ca0</ncml:id></ncml:lt>
<ncml:id>ca0</ncml:id>
<ncml:id>comp95:ca</ncml:id></ncml:apply>
<ncml:id>comp95:ca_beta</ncml:id></ncml:mul></ncml:neg></ncml:sum></ncml:expr></ncml:rate>
<ncml:asgn id="comp95:cac">
<ncml:expr>
<ncml:apply id="if">
<ncml:lt>
<ncml:id>comp95:ca</ncml:id>
<ncml:id>ca0</ncml:id></ncml:lt>
<ncml:id>ca0</ncml:id>
<ncml:id>comp95:ca</ncml:id></ncml:apply></ncml:expr></ncml:asgn>
<ncml:output id="comp95:cac"></ncml:output></ncml:component>
<ncml:component name="comp96" type="membrane-capacitance">
<ncml:const id="comp96:C_m">
<ncml:expr>0.001</ncml:expr></ncml:const>
<ncml:output id="comp96:C_m"></ncml:output></ncml:component></ncml:model>
```
